# Chromosome-level genome assembly of *Murraya paniculata* sheds light on biosynthesis of floral volatiles

**DOI:** 10.1186/s12915-023-01639-6

**Published:** 2023-06-20

**Authors:** Tianyu Yang, Xin Yin, Haotong Kang, Danni Yang, Xingyu Yang, Yunqiang Yang, Yongping Yang

**Affiliations:** 1 School of Life Science, Yunnan University, Kunming, 650500 China; 2 Key Laboratory for Plant Diversity and Biogeography of East Asia, Kunming Institute of Botany, Chinese Academy of Science, Kunming, 650201 China; 3 Plant Germplasm and Genomics Center, Kunming Institute of Botany, Chinese Academy of Sciences, Kunming, 650201 China; 4 Institute of Tibetan Plateau Research at Kunming, Kunming Institute of Botany, Chinese Academy of Sciences, Kunming, 650201 China; 5 University of Chinese Academy of Sciences, Beijing, 100049 China; 6 Key Laboratory of Plant Resources Conservation and Utilization, College of Biological Resources and Environmental Sciences, Jishou University, Jishou, 416000 China

**Keywords:** *Murraya paniculata*, *Citrus* species, Comparative genomics, Transposon, Flower volatiles, Phenylacetaldehyde

## Abstract

**Background:**

*Murraya paniculata* (L.) Jack, commonly called orange jessamine in the family Rutaceae, is an important ornamental plant in tropical and subtropical regions which is famous for its strong fragrance. Although genome assemblies have been reported for many Rutaceae species, mainly in the genus *Citrus*, full genomic information has not been reported for *M. paniculata*, which is a prerequisite for in-depth genetic studies on *Murraya* and manipulation using genetic engineering techniques. Here, we report a high-quality chromosome-level genome assembly of *M. paniculata* and aim to provide insights on the molecular mechanisms of flower volatile biosynthesis.

**Results:**

The genome assembly with a contig N50 of 18.25 Mb consists of 9 pseudomolecules and has a total length of 216.86 Mb. Phylogenetic analysis revealed that *M. paniculata* diverged from the common ancestor approximately 25 million years ago and has not undergone any species-specific whole genome duplication events. Genome structural annotation and comparative genomics analysis revealed that there are obvious differences in transposon contents among the genomes of *M. paniculata* and *Citrus* species, especially in the upstream regions of genes. Research on the flower volatiles of *M. paniculata* and *C. maxima* at three flowering stages revealed significant differences in volatile composition with the flowers of *C. maxima* lacking benzaldehyde and phenylacetaldehyde. Notably, there are transposons inserted in the upstream region of the *phenylacetaldehyde synthase* (*PAAS*) genes *Cg1g029630* and *Cg1g029640* in *C. maxima*, but not in the upstream region of three *PAAS* genes *Me2G_2379*, *Me2G_2381*, and *Me2G_2382* in *M. paniculata*. Our results indicated that compared to the low expression levels of *PAAS* genes in *C. maxima*, the higher expression levels of the three *PAAS* genes in *M. paniculata* are the main factor affecting the phenylacetaldehyde biosynthesis and causing the content difference of phenylacetaldehyde. The phenylacetaldehyde synthetic activities of the enzymes encoded by *M. paniculata PAAS* genes were validated by in vitro analyses.

**Conclusions:**

Our study provides useful genomic resources of *M. paniculata* for further research on Rutaceae plants, identifies new *PAAS* genes, and provides insights into how transposons contribute to variations in flower volatiles among *Murraya* and *Citrus* plants.

**Supplementary Information:**

The online version contains supplementary material available at 10.1186/s12915-023-01639-6.

## Background

*Murraya paniculata* (L.) Jack, commonly called orange jessamine and synonymous with *Murraya exotica*, belongs to the genus *Murraya* in the family Rutaceae [1] (Additional file 1: Fig. S1). This ornamental plant is commonly used in landscaping and is now cultivated worldwide, including in Africa, America, the Caribbean, South America, Europe, and Oceania. Its leaves and flowers can be used to extract essential oils or for medicinal purposes, and the roots and stems also have a variety of uses [2]. *M. paniculata* is described in the Chinese pharmacopoeia as having anti-inflammatory, anti-biotic, and analgesic properties. A hot water extract of dried roots or stems can be used as an ecbolic with full-term deliveries for pregnant women in China. The dried bark and fruit are used in South East Asia as an astringent, to reduce fever, and to treat dysentery [3]. The stem bark is used in India for the treatment of coughs, hysteria, and rheumatism, while a paste of the leaf mixed with turmeric powder is applied to soothe fractured bones [4]. The volatiles collected from *M. paniculata* could attract *Diaphorina citri*, a vector of the bacterial causative agent of Huanglongbing [5] whereas *M. paniculata* and *M. koenigii* are reported to be resistant to the disease [6, 7].

As opposed to its use as a traditional medicine in the past, *M. paniculata* is now more likely to be sold in nurseries and stores as an ornamental plant. Its flowers have a strong fragrance that enhances its ornamental value. Studies have shown that flowers of *M. paniculata* follow a nocturnal/crepuscular pattern of blooming [8]. The volatile compounds, which are usually emitted at night, mainly include benzenoids, terpenoids, indoles, phenylacetaldehyde, and methyl palmitate [8]. The flowering time of *M. paniculata* can be divided into three stages; early, mid, and late, according to the dynamics of compounds emitted during the day. In the early flowering stage, the main components of the flower volatiles are phenolic compounds. At the mid-flowering stage when the flowers are fully open, terpenoids are the main volatiles. At the late-flowering stage, large amounts of phenylacetaldehyde are emitted. Each metabolite in the flower volatiles released during the flower’s life cycle is believed to have a specific function [9, 10]. For example, benzenoids attract pollinators, while terpenoids have the dual effect of attracting and discouraging insects from visiting flowers [11]. The emission of large amounts of phenylacetaldehyde during the daytime can deter possible nectar thieves that visit during the late-flowering stage [8].

The genus *Murraya* and *Citrus* both belong to the subfamily Aurantioideae of the family Rutaceae. However, the components of flower volatiles differ among *M. paniculata* and some *Citrus* plants, including *C. maxima*, *C. sinensis*, *C. limon*, and *C. aurantium* [8, 12, 13]. The volatiles of most *Citrus* plants mainly consist of linalool, β-myrcene, α-myrcene, limonene, (E)-ocimene, methyl anthranilate, and indole, but benzaldehyde and phenylacetaldehyde are missing in *Citrus* flower volatiles while they are enriched in the flower volatiles of *M. paniculata* [8, 12, 13]*.* The molecular basis for the biosynthesis of these compounds and the reason for the significant differences in volatiles between *Citrus* and *Murraya* are still unknown.

Here, we report the first chromosome-level reference genome of *M. paniculata* with a total assembly length of 216.87 Mb of 9 chromosomes and a contig N50 of 18.25 Mb. Comparative genomic analyses with other published Aurantioideae genomes revealed that transposon expansion is responsible for the different genome sizes between *M. paniculata* and the other Aurantioideae species whose genome size were reported to be in a range of 301.39 Mb (*C. clementina*) to 406.06 Mb (*C. medica*) [14–20] (http://citrus.hzau.edu.cn/statistics.php#genomeInfo). Furthermore, the flower volatiles analysis and transcriptome analysis in *M. paniculata* and *C. maxima* at different developmental periods showed how transposon polymorphism has affected the expression of genes encoding phenylacetaldehyde synthase. The reference genome of *M. paniculata* is a valuable genomic resource for further research on Rutaceae plants. Studies on floral volatiles and comparative genomics analysis reveal that transposon insertion may affect plant volatile biosynthesis and provide insights into the molecular mechanism of floral volatile biosynthesis in *M. paniculata*.

## Results

### Genome assembly and annotation

**Fig. 1:**
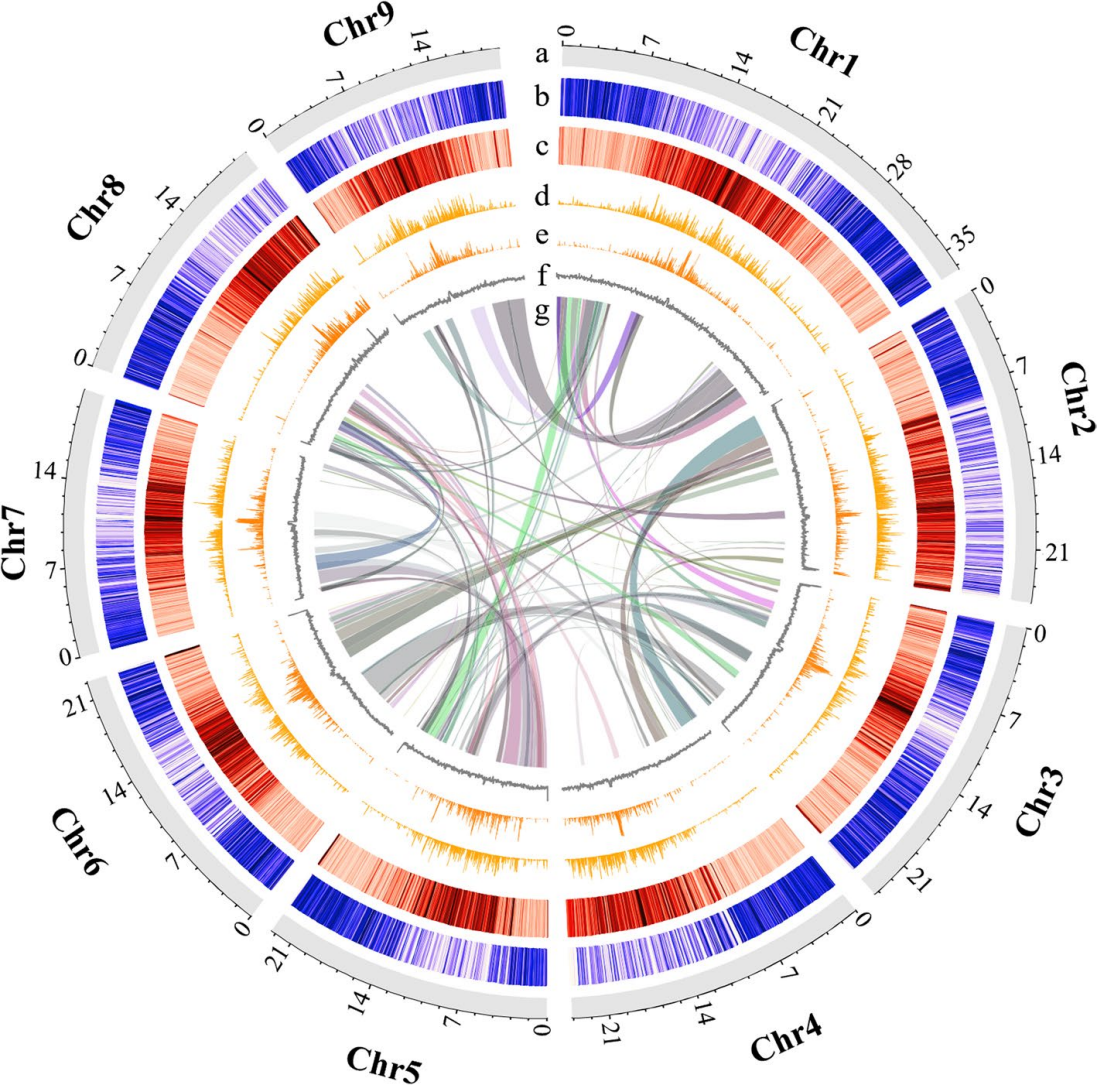
*M. paniculata* genomic landscape. (a) chromosomes, (b) gene density, (c) repeat density, (d) Copia distribution, (e) Gypsy distribution, (f) GC content, (g) collinear blocks. The density showing in heatmaps and histograms was calculated using 500 kb sliding windows. The darker the colour in the heatmaps, the higher the density

The genome size of *M. paniculata* is estimated to be about 256.76 Mb, with a heterozygosity rate of 0.44% based on *K*-mer analysis (*K* = 21) (Additional file 1: Fig. S2) using 8.74 Gb NGS short reads. To obtain a high-quality, chromosome-level genome assembly, 34.14 Gb ONT long reads (142 × coverage depth), 18.57 Gb paired-end NGS short reads (73 ×), and 41.78 Gb paired-end Hi-C reads (163 ×) were generated by different sequencing platforms (Additional file 2: Table S1). Firstly, the ONT long reads and NGS short reads were used for primary assembly, error correction, and polishing, finally generating an assembly of 216.87 Mb with a contig N50 of 18.25 Mb. Using 208.27 million Hi-C reads (Additional file 2: Table S1), contigs in the polished assembly were successfully ordered, oriented, and clustered into 9 pseudomolecules (Additional file 1: Fig. S3) (Additional file 2: Table S2). About 64.06 Mb repeat sequences were identified, accounting for 29.54% of the assembly, including a large amount of long terminal repeat (LTR) retrotransposons with a numerous of Ty3-Gypsy (7.29%) and Ty1-Copia (5.37%) (Fig. 1; Table 1; Additional file 2: Table S3). Using a combination of de novo prediction, homology alignment prediction, and transcriptome-based prediction, we predicted 23,548 protein-coding genes in the *M. paniculata* genome (Fig. 1; Table 1; Additional file 2: Table S4). Approximately 70.93% (16,703) of the protein-coding genes were functionally annotated by Swiss-Prot (Additional file 2: Table S4). In addition, 163 miRNAs, 246 rRNAs, and 481 tRNAs were identified (Table 1; Additional file 2: Table S5). The completeness was 98.24% and 98.50% when BUSCO was run in the genome and protein mode, respectively (Additional file 2: Table S6 and Table S7).

**Table 1:** Summary of genome assembly and annotation for the *M. paniculata*

**Primary genome assembly statistics**	
Total length (Mb)	216.87
Number of contigs	18
Contig N50 (Mb)	18.25
Contig L50	5
Contig N90 (Mb)	7.93
Contig L90	13
Max contig size (Mb)	25.11
**Pseudomolecules statistics**	
Number of Pseudomolecules	9
Scaffold N50 (Mb)	23.92
Scaffold N90 (Mb)	20.67
GC content (%)	34.23
Max Scaffold size (Mb)	36.72
Total length (Mb)	216.31
**Annotation statistics**	
Annotated protein-coding genes	23,548
miRNA	163
rRNA	246
tRNA	481
Repeat sequence length (Mb)	64.06

### Comparative genomics analysis

**Fig. 2:**
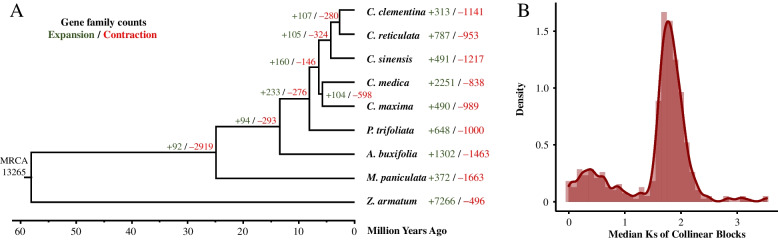
Comparative genomic analysis of *M. paniculata*. **A** Phylogenetic tree constructed using coding sequences of single-copy gene families among 9 Aurantioideae species. Divergence times were estimated by MCMCTree. Support rates were calculated by RAxML-NG with GTR + G model and 200 bootstraps. All nodes have a bootstraps support percentage of 100. The green and red numbers on the tree or next to the species label indicate the expansion and contraction gene family counts. **B** Density distributions of collinear block Ks (synonymous substitutions per synonymous site) median of *M. paniculata*

To investigate the phylogenetics of *M. paniculata*, its protein sequences and those of *C. clementina*, *C. reticulata*, *C. sinensis*, *C. medica*, *C. maxima*, *Poncirus trifoliata*, *Atalantia buxifolia*, and *Zanthoxylum armatum* as the outgroup were collected for comparative genomics analysis. In total, 4995 single-copy gene families were identified and used for phylogenetic tree construction and estimation of divergence time (Fig. 2A). The phylogenetic tree revealed that *M. paniculata* diverged from the common ancestor approximately 25 (17.5–30.7) million years ago (MYA) (Fig. 2A). Expansion and contraction of gene families was also estimated from the tree. A total of 372 significantly expanded gene families (*P* < 0.05) and 1663 contracted gene families (*P* < 0.05) were detected in *M. paniculata*. KEGG pathway enrichment analysis using the genes in expanded gene families showed that they are enriched in some metabolic pathways such as “amino acid metabolism”, “phenylpropanoid biosynthesis”, and “sesquiterpenoid and triterpenoid biosynthesis” (Additional file 3). The homologous gene pairs in *M. paniculata* were collected for whole genome duplication (WGD) analysis. The synonymous substitutions per site (Ks) distance of these gene pairs revealed that *M. paniculata* has not undergone any species-specific WGD events after the shared ancient WGD in *M. paniculata* and *C. sinensis* (Ks 1.4–1.5) (Fig. 2B; Additional file 1: Fig. S4) [19]. These genomes of *M. paniculata*, *C. sinensis*, *C. medica*, and *P. trifoliata* show high collinearity (Additional file 1: Fig. S5). However, the genome size and transposable elements (TEs) length of *M. paniculata* are smaller than the other three species (Additional file 1: Fig. S6; Additional file 2: Table S8 and Table S9).

To further investigate the difference in the repeat sequence among these species, the TEs identified in these four species were classified in more detail. The retrotransposons in *M. paniculata* included 28.24 Mb of LTR sequences, mainly Gypsy and Copia, accounting for approximately 12.66% of the genome; while the DNA transposons mainly consisted of the terminal inverted repeat (TIR) superfamilies CACTA, Mutator, PIF-Harbinger, Tc1-Mariner, and hAT [21]. The number of LTRs in the *P. trifoliata*, *C. maxima* and *C. sinensis* genomes was 1.42-times, 2.54-times, and 1.71-times than that in the *M. paniculata* genome, respectively (Additional file 1: Fig. S7). We calculated the distribution density of LTRs and TIRs in the 10-kb range upstream and downstream of genes’ coding regions with 100-bp sliding windows (Additional file 1: Fig. S8). The percentage of genes with LTRs inserted nearby was significantly lower in the genome of *M. paniculata* than in the genomes of the other three species (Additional file 1: Fig. S8). The percentage and distribution of TIRs differed slightly among the four species (Additional file 1: Fig. S8). A significant expansion of the transposase gene family was detected in the genomes of *C. sinensis* and *C. maxima* (Additional file 1: Fig. S9). Next, genes from these four species with LTR insertions in the 3-kb upstream region were collected for KEGG pathway enrichment analysis separately (Additional file 4), which showed that the genes from different species with LTR inserted nearby may be similarly involved in several metabolites biosynthesis pathways. These results imply that the LTR insertions may affect the metabolite biosynthesis in these species.

### Differences in flower volatile components between *M. paniculata* and *C. maxima*

**Fig. 3:**
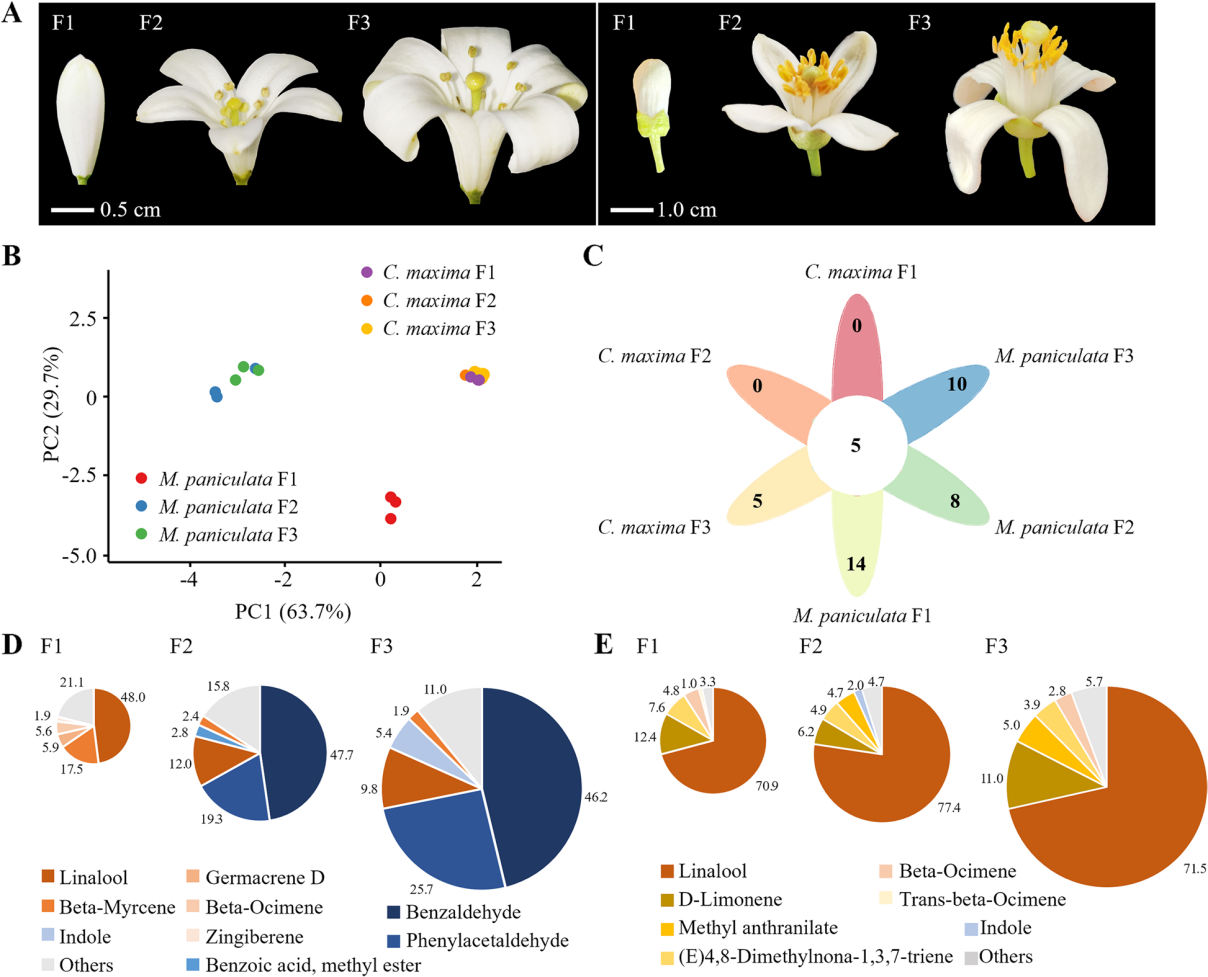
Analysis of flower volatiles of *M. paniculata* and *C. maxima*.** A** Flower morphology of *M. paniculata* (left) and *C. maxima* (right) at different flowering stages. **B** Principal component analysis (PCA) of flower volatile components at different flowering stages. Scale of the axis is relative distance. Dots with different colours represent different groups of samples. **C** Venn diagram showing shared or unique types of volatiles among different flowering stages of *M. paniculata* and *C. maxima*. **D **and** E** Relative percentage content of volatiles out of total flower volatiles at different flowering stages of *M. paniculata* (**D**) and *C. maxima *(**E**). Data for volatile contents statistics were obtained from three replicates of flowers with similar growth status. Area of each sector represents the relative abundance of corresponding volatile compound calculated by GC–MS peak area (Additional files 5 and 6)

To explore the volatiles differences of flowers between *Murraya* and *Citrus*, we identified and quantified the flower volatiles of *M. paniculata* and *C. maxima* at different flowering stages (F1: early flowering, F2: mid flowering and F3: late flowering) (Fig. 3A) by headspace solid-phase microextraction (HS-SPME) and gas chromatography-mass spectrometry (GC–MS) (Additional files 5 and 6). As the principal component analysis (PCA) showed, the volatile compounds at the F2 and F3 flowering stages were separated from those at the F1 flowering stage in *M. paniculata*, whereas the flower volatiles at all three flowering stages of *C. maxima* were clustered together (Fig. 3B). There were five shared volatile compounds at all flowering stages of *M. paniculata* and *C. maxima*, but more compounds unique to *M. paniculata* flowers (Fig. 3C). In the F1 flowering stage of *M. paniculata*, the main volatile compounds were linalool (48.0%) and beta-myrcene (17.5%). However, we found a dramatic change in volatiles composition in the F2 and F3 flowering stages of *M. paniculata*, with benzaldehyde (47.7% and 46.2%, respectively) and phenylacetaldehyde (19.3% and 25.7%, respectively) being the main volatile components (Fig. 3D). The main volatile components of *C. maxima* flower volatiles were linalool and D-limonene at all flowering stages (Fig. 3E). The comparison of the flower volatile components between *C. maxima* and *M. paniculata* showed that *C. maxima* lacks phenylacetaldehyde and benzaldehyde, which are the main volatile compounds in flower volatiles of *M. paniculata* at the F2 and F3 stages. Considering that many genes involved in metabolites biosynthesis pathways have TE insertions nearby (Additional file 4), we speculated that the volatiles difference between *M. paniculata* and *C. maxima* may be related to TE insertion around genes in relevant pathways.

### TEs insertions may lead to differential *PAAS *expression in *M. paniculata* and *C. maxima*

**Fig. 4:**
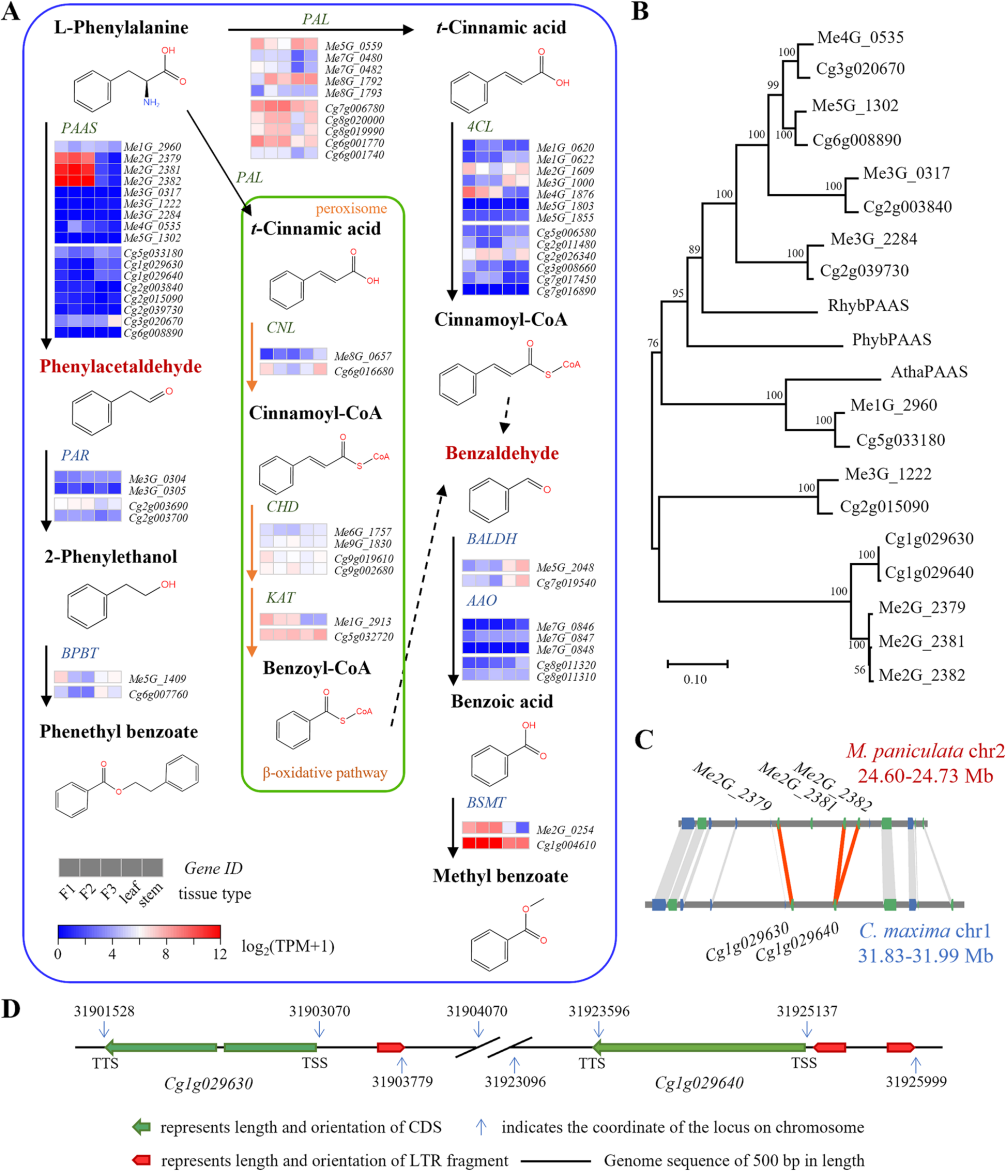
Analysis of genes involved in benzaldehyde and phenylacetaldehyde biosynthesis and metabolic pathway.** A** Genes involved in benzaldehyde and phenylacetaldehyde biosynthesis and metabolic pathway with heatmaps showing the relative expression levels in flowers from three flowering stages, leaves, and stems. TPMs were obtained from the mean values of three replicates (Additional file 7). Gene expression levels are normalised and represented as log_2_(TPM + 1). Blue, low expression levels; red, high expression levels. **B** Protein neighbour-joining tree of *PAAS* genes in f *M. paniculata* and *C. maxima* with outgroups of RhybPAAS (*Rosa hybrid*; Uniprot entry: Q0ZS27), PhybPAAS (*Petunia hybrida*; Uniprot entry Q0ZQX0), and AthaPAAS (*A. thaliana*; Uniprot entry: Q8RY79) constructed with 1000 bootstrap replicates. **C** Gene collinearity analysis between *Me2G_2379*, *Me2G_2381,* and *Me2G_2382* in *M. paniculata* and *Cg1g029630*, *Cg1g029640* in *C. maxima*. **D** Overview of LTR insertions upstream *PAAS* genes *Cg1g029630* and *Cg1g029640* in *C. maxima.* Black line represents genome sequence; green bold arrow represents length and orientation of CDS; red bold arrow represents length and orientation of LTR fragment. TSS: transcription start site; TTS: transcription termination site

To validate the hypothesis and find evidence for the effect of TE insertions on, the genes involved in the benzaldehyde and phenylacetaldehyde biosynthesis and metabolism pathway were identified in *M. paniculata* and *C. maxima*, respectively (Fig. 4A). These genes belonged to 11 gene families. Compared with *C. maxima*, *M. paniculata* had one extra member in the *PAL*, *PAAS*, *4CL*, and *AAO* gene family. Three tandemly repeated *PAAS* genes in *M. paniculata*, namely *Me2G_2379*, *Me2G_2381*, and *Me2G_2382*, caught our attention, whose relative expression levels were significantly higher than those of their two homologues in *C. maxima* (*Cg1g029630* and *Cg1g029640*) (Fig. 4A and B; Additional file 7). Genome structural analysis revealed that there are no LTR insertions in the upstream region of the three *PAAS* genes of *M. paniculata*, while insertions with the same LTR fragment were observed in the upstream promoter regions of *Cg1g029630* and *Cg1g029640* (Fig. 4C and D; Additional file 1: Fig. S10), which may affect the expression levels of *Cg1g029630* and *Cg1g029640*, thus leading to the difference of phenylacetaldehyde content in the flowers of *M. paniculata* and *C. maxima*. Similar LTR insertions were also observed in the promoter regions of corresponding *PAAS* genes in *C. sinensis* (Additional file 1: Fig. S10 and Fig. S11).

### Biosynthesis of phenylacetaldehyde by three master-effect *PAAS *genes in *M. paniculata*

**Fig. 5:**
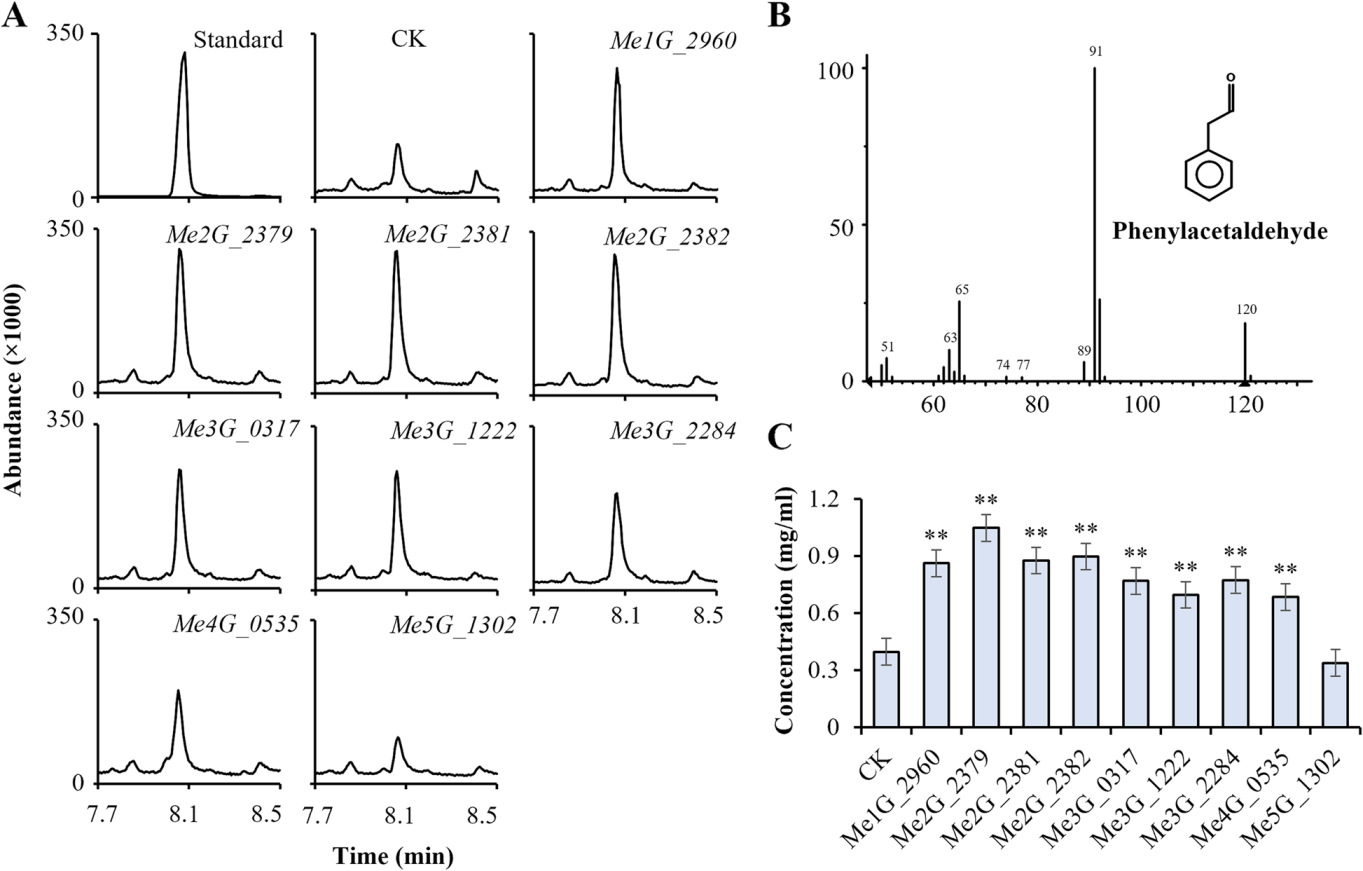
Phenylacetaldehyde analysis using PAAS heterologously expressed in *Escherichia coli*.** A** GC–MS results showing that *Me1G_2960*, *Me2G_2381*, *Me3G_0317*, *Me3G_1222, Me3G_2284*, *Me4G_0535*, and *Me5G_1302* encode enzymes that are functional when expressed in *Escherichia coli*. Extract ion chromatogram shows products produced by strains expressing target genes or the empty vector control (CK). Peaks at retention time about 8 min represent phenylacetaldehyde. X-axis represents retention time; Y-axis represents relative abundance. **B** Mass spectrum of phenylacetaldehyde (molecular ion m/z 91).** C** Concentration of phenylacetaldehyde produced by products of 9 *PAAS* genes. Data (mean values ± SEs) were obtained from three replicate experiments (Student’s *t*-test, *P < 0.05, **P < 0.01)

We identified a total of 9 *PAAS* genes in *M. paniculata*, but only some of them were highly expressed in the flower in the flowering stages. Thus, we verified and compared the catalytic activity of the products of these *PAAS* genes to clarify their role in phenylacetaldehyde biosynthesis. The optimised full-length CDS of these *PAAS* genes (Additional file 2: Table S10) were transferred into the *Escherichia coli* expression strain Rosetta (DE3), and the phenylacetaldehyde content in the assay mixture was determined by GC–MS. The proteins encoded by most *PAAS* genes were able to catalyse the production of phenylacetaldehyde (Fig. 5; Additional file 8). Furthermore, kinetic characterisation of purified recombinant PAAS revealed an apparent Km for L-phenylalanine (L-Phe) of 1.13 ± 0.31 mM − 36.59 ± 4.35 (mean ± S.E., *n* = 3). The PAAS Kcat were 0.96 ± 0.26 × 10^−3^ − 34.60 ± 0.2612.63 × 10^−3^ min^−1^ (mean ± S.E., *n* = 3) (Additional file 1: Fig. S12; Additional file 2: Table S11). The proteins encoded by *Me2G_2379*, *Me2G_2381*, and *Me2G_2382* showed very strong catalytic activity to produce phenylacetaldehyde. Combined with the results of transcriptome analysis, these three genes likely encode the main enzymes involved in the biosynthesis of phenylacetaldehyde in *M. paniculata* flowers.

## Discussion

Flower volatile compounds have various roles, they attract insects for pollination, improve plant reproduction and adaptability, and act as a language to communicate and interact with the surrounding environment [22, 23]. As ornamental plants with strong flower aroma, the flower volatiles of *M. paniculata* differ from those of *Citrus* plants in Aurantioideae. The genetic basis of the diversity of specialised metabolites can be illustrated by high-quality genome sequencing [24]. Most of the Rutaceae genomes published so far are in the genus *Citrus*, including *C. clementina*, *C. reticulata*, *C. maxima*, *C. sinensis*, and *C. medica*, but genomic information was not available for the genus *Murraya*. In this study, we presented a high-quality reference genome of *M. paniculata*, which is the first chromosome-level genome assembly in *Murraya* plants. *M. paniculata* diverged from Rutaceae about 25 MYA. No large-scale variation in chromosome structure was detected in the *M. paniculata* genome compared with that of *C. sinensis*. Besides the core eudicot shared γ triplication event, *M. paniculata* has not undergone any additional species-specific WGD events, consistent with *C. sinensis* [19]. Genomes of *M. paniculata* and other published *Citrus* plants have a high degree of genetic collinearity, implying that different characters between *M. paniculata* and *Citrus* may be not due to large-scale chromosome structural variations.

TEs are major component of plant genomes, their activity and diversity can influence the genome size and structure [25, 26]. Thus, considering the differences in genome size among *M. paniculata* and some representative species in Aurantioideae subfamily, we analysed the TEs in their genomes. The results showed that there are significantly more TEs in the *C. sinensis* and *C. maxima* genomes than in the *M. paniculata* genome. In particular, the numbers of LTRs distributed near protein-coding genes are about two-fold higher in *C. sinensis* and *C. maxima* than in *M. paniculata*. Several studies have shown that LTRs tend to be inserted near genes with functions in resistance and development, driving divergence among related species [27, 28]. We found that genes with TEs insertions in their upstream 3-kb regions are mainly enriched in several types of metabolites biosynthesis pathways in *M. paniculata* and *C. maxima* whereas the GC–MS analysis revealed notable differences in flower volatile compounds between them in three flowering stages. Thus, we hypothesised that the differences in flower volatiles may be related to TE insertions in the vicinity of genes encoding biosynthetic or metabolic enzymes in these plants [23].

The insertion of TEs in genomic regions can seriously affect the regulation of neighbouring genes’ expression, resulting in altered traits. In *Arabidopsis thaliana* and *A. lyrata*, TEs reduce the average expression levels of adjacent genes because of interference with *cis*-regulatory elements, and these effects differ between the two species [27]. Genomic studies on apple and Sicilian blood orange revealed LTR insertions influence the expression of adjacent genes [29–31]. In this study, we found LTR inserted upstream *C. maxima PAAS* genes (*Cg1g029630* and *Cg1g029640*) (Fig. 4D), which may be associated with their relatively low expression levels in flowers. Three homologous *M. paniculata PAAS* genes (*Me2G_2379*, *Me2G_2381*, and *Me2G_2382*) that have no LTR insertions nearby show higher transcript levels during flower development. The proteins of these three genes were shown to have high phenylacetaldehyde synthesis activity (Fig. 5C). Considering the fact that phenylacetaldehyde is one of the main components of the flower volatiles of *M. paniculata*, the difference between *M. paniculata* and *C. maxima* flower volatiles may be largely caused by these TE insertions adjacent to *C. maxima PAAS* genes*,* which results in the lack of phenylacetaldehyde in *C. maxima* flowers. More experimental evidence and bioinformatic analysis are required to further investigate the molecular mechanisms underlying the differences in volatiles.

## Conclusions

The study presented a high-quality chromosome-level genome assembly of *M. paniculata*. Obvious differences in transposon contents were detected in the genomes among *M. paniculata* and other Aurantioideae species, especially in the upstream regions of genes, which may affect metabolites biosynthesis*.* Furthermore, three genes (*Me2G_2379*, *Me2G_2381*, and *Me2G_2382*) with strong synthetic activity for phenylacetaldehyde were newly identified in *M. paniculata*, whereas the expressions of two homologues of these genes in *C. maxima* may be affected by LTR insertions which led to the lack of phenylacetaldehyde in flowers of *C. maxima*. The study proposed how transposons impact on the composition of flower volatiles among species in *Murraya* and *Citrus*. These genomic resources of *M. paniculata* will contribute to further research on species in Rutaceae.

## Methods

### Plant materials

Fresh healthy leaves were harvested from an individual plant of *M. paniculata* at Xishuangbanna Tropical Botanical Garden, Chinese Academy of Sciences (21°55′11″N, 101°15′27″E) in April 2021 and immediately frozen in liquid nitrogen, then stored at − 80 °C until DNA extraction. Leaves, stems, and flowers from *M. paniculata* were also sampled for RNA-seq. Flowers at three different flowering stages were collected from different *M. paniculata* and *C. maxima* individuals for volatile identification and transcriptome analysis at Xishuangbanna Tropical Botanical Garden in March 2022.

### Library construction and sequencing

For ONT sequencing, high-quality genomic DNA was extracted, size-selected to prepare the ONT library, and sequenced on the PromethION platform (Oxford Nanopore Technologies, Oxford, UK). For whole genome sequencing on the NGS platform, a paired-end library with an insert size of 350 bp was constructed according to the manufacturer’s protocol. For Hi-C sequencing, fresh young leaves were fixed in formaldehyde (1 g leaf material per 100 mL). Chromatin was cross-linked and digested using the restriction enzyme *Dpn*II. Then the DNA sample was biotin labelled and ligated. After shearing and size-selecting, fragments containing biotin were captured to construct the paired-end library. For RNA sequencing, the total RNA was extracted from mixed leaf, stem, and flower tissues using RNAiso Plus (Qiagen, Hilden, Germany) according to the manufacturer’s protocol and libraries were prepared using the TruSeq RNA Sample Preparation kit. The quantifications of these paired-end libraries were performed using a Qubit 3.0 fluorometer (Thermo Fisher Scientific Inc., Waltham, MA, USA) and the quality assessments were carried out by an Agilent 2100 instrument (Agilent, Palo Alto, CA, USA). Qualified libraries were then sequenced using the DNBSEQ-T7 platform (MGI, Shenzhen, China) with a layout of 150 bp. All paired-end raw reads were filtered by fastp v0.23.2 [32] (-f 5 -t 5 -n 0 -l 140).

### Genome assembly and pseudomolecule construction

Raw reads from ONT sequencing were error corrected and trimmed using Canu v2.1.1 [33] (-nanopore -correct -trim) and the longest 40 × reads were retained to generate the draft assembly by NextDenovo v2.4.0 [34] and polished with ONT and NGS reads using NextPolish [35] (task = best). Redundancies were removed using Purge_Dups [36]. Bwa-mem2 v2.2 [37] was used to align the clean Hi-C reads to the draft assembly. A valid Hi-C links file was obtained using juicer v1.6 [38]. Then the links file was processed by 3D-DNA v201008 [39] to order, orient, and cluster the contigs automatically followed by manual validation and adjustments by juicebox v1.11.08 [40]. The Hi-C contact heatmap was generated using the 3D-DNA visualization module and juicebox.

### Genome annotation

A high-quality non-redundant repeat library of the *M. paniculata* genome was generated using the Extensive de novo TE Annotator (EDTA) pipeline [41]. The TEs were further classified by TEsorter [42] and intact transposase sequences were extracted simultaneously for tree building using iTOL [43]. Based on the.out file, the EDTA pipeline summarised the TEs’ counts and proportions. The same pipeline was used to process the genomes of *P. trifoliata*, *C. maxima*, and *C. sinensis*. The classified repeat library was used to softmask the genome by RepeatMasker (http://www.repeatmasker.org) [44]. The structural annotation for protein-coding genes was based on de novo prediction, homologous protein alignment, and RNA-seq-based predictions using BRAKER v2.1.6 [45] with the softmasked genome. The RNA-Seq reads were mapped to the genome using HISAT2 v2.2.1 [46] with default parameters. The proteins in OrthoDB v10 [47] were used for protein-based gene prediction*.* Protein hints were generated using ProtHint v2.6.0 [48]. De novo gene prediction was carried out using protein alignment data and RNA-seq data with AUGUSTUS v3.4.0 [49] and GeneMark-ES Suite version 4.69_lic [50]. TSEBRA v1.0.3 [51] was used to merge the prediction results. The annotation file was modified and formatted by MAKER v3.01.04 [52] and EVidenceModeler [53]. Gene function annotation was conducted using both eggNOG-mapper [54] online (http://eggnog-mapper.embl.de/) and BLAST-based methods. The best alignments were obtained by searching against the NR and SwissProt databases using diamond v2.0.11.149. The completeness of genome assembly and annotation was assessed using Benchmarking Universal Single-Copy Orthologs (BUSCO) [55] with eudicots_odb10.

### Phylogenetic analysis

Orthogroups were identified using OrthoFinder v2.3.12 [56] with default parameters taking protein sequences from *C. clementina*, *C. reticulata*, *C. sinensis*, *C. medica*, *C. maxima* [20], *P. trifoliata*, *A. buxifolia*, and *Z. armatum* as inputs [57, 58]. Protein sequences from single-copy orthogroups were collected and aligned using MAFFT v7.313 with the G-INS-i strategy [59]. The alignments of single-copy orthogroups were concatenated to generate a super alignment matrix, and a maximum-likelihood tree was constructed using the GTR + G model with 200 bootstraps by RAxML-NG. Divergence times were estimated using MCMCTree in the PAML v4.10.6 [60]. Analyses were run for 100,000 generations with a burn-in of 400,000 iterations and the HKY85 model. The fossil calibration times used to estimate divergence time (13.5–30 MYA between *Murraya* and *Atalantia* and 25.1–69.9 MYA between *Murraya* and *Zanthoxylum*) were obtained from the TimeTree of Life online in April 2023 [61]. All MCMCTree calculations were run twice to ensure convergence. The expansion and contraction of orthogroups were calculated using CAFE5 [62] under a random birth-and-death model using the OrthoFinder results as input. The gene synteny and gene duplication analyses were performed using JCVI with the MCscan pipeline [63]. Syntenic blocks and synonymous nucleotide substitutions (Ks) were determined and calculated from protein sequence alignments using the NG86 model based on the homologous gene pairs identified by WGDI [64]. The WGD and speciation events were inferred from paralogous and orthologous pairs of Ks distribution peaks, respectively.

### Flower volatile compounds analysis

The volatile compounds of the flowers were analysed by HS-SPME coupled with GC–MS. Each sample was quickly placed in a wet glass vessel (50 mL) and sealed with paraffin film. The SPME fibre (50/30 μM DVB/CAR/PDMS) was exposed to the inflorescence for 30 min to extract the volatile components in an air-conditioned laboratory at 25 °C ± 3 °C. After the adsorption period, the fibre head was removed and introduced into the heated injector port of the GC for desorption at 250 °C for 1 min. Subsequent analysis was carried out using an Agilent GC–MS (6890 GC-5973N MSD, Agilent, Santa Clara, CA, USA). The carrier gas (He) was supplied at a constant rate of 1.0 mL/min. The mass detector conditions for MS were as follows: source temperature of 250 °C, electronic impact (EI) mode at 70 eV, with a speed of 4 scans/s over the mass range m/z 33–450 amu in a 1-s cycle. Compounds were auto-matched with mass spectra in the NIST98 database through Chem-Station (Agilent). The normalised peak area measurements were used to calculate the percentage of each flower volatile component.

### Candidate genes identification

Protein BLASTN and Pfam annotation were used to identify genes involved in benzaldehyde and phenylacetaldehyde biosynthesis and metabolic pathways in *M. paniculata* and *C. maxima*. The searching queries were the functionally validated proteins including RhybPAAS (*Rosa hybrid*; Uniprot entry: Q0ZS27), PhybPAAS (*Petunia hybrida*; Uniprot entry Q0ZQX0), and AthaPAAS (*A. thaliana*; Uniprot entry: Q8RY79) [65] obtained from Swiss-Prot database. Genes with high homology and similar domains were identified as candidates.

### Calculation of gene relative expression level

The raw RNA-seq reads were first filtered by fastp as described above and then mapped to the reference genome using HISAT2 (–very-sensitive –dta). Raw count for each transcript was tallied by featureCounts v2.0.3 [66] and normalised by TPM (Transcripts Per kilobase of exon model per Million mapped reads) values using an R script. The heatmap was generated using TBtools [67].

### Enzyme activity analysis

According to the amino acid sequences, codon-optimised *PAAS* genes from *M. paniculata* were synthesised and then pET28a-PAAS recombinant plasmids (Additional file 2: Table S12) were transferred into *E. coli* Rosetta (DE3) competent cells. The control was transformed with the plasmid without an insert. The cells were grown in LB medium containing 50 μg/mL kanamycin at 37 °C to a culture density of OD600 = 0.5. Then, the expression of PAAS proteins was induced by the addition of IPTG to a final concentration of 0.3 mM. After a 13-h incubation at 18 °C, the cells were harvested by centrifugation and resuspended in lysis buffer containing 0.02 M phosphate buffer (pH 7.4), 0.5 M NaCl, 2% (v/v) glycerol, 0.2 mM pyridoxal 5-phosphate (PLP), and 1 mM PMSF. After removing cell debris, proteins were purified by affinity chromatography on nickel nitrilotriacetic acid-agarose (0.5-mL bed volume) [65].

An in vitro reaction was established with the above-described purified proteins. The time dependence was conducted in reactions of PAAS (5 μg), 5 mM L-Phe, 50 mM Tris–HCl (pH 8.5), 0.2 mM PLP, and 0.1 mM EDTA in a final volume of 50 µL, kept at 28 °C for 10 min, 20 min, 30 min, 40 min, 50 min, 60 min, respectively. Kinetic studies were performed with PAAS (4 mg/mL), L-Phe (2.7 mM, 5.4 mM, 10.8 mM, 21.6 mM, 43.3 mM, respectively), 50 mM Tris–HCl (pH 8.5), 0.2 mM PLP, and 0.1 mM EDTA in a final volume of 50 µL, incubating at 28 °C for 30 min. After incubation at 28 °C, the reaction was stopped by adding 5 μL 10 M NaOH, and then 250 μL ethylacetate was added to extract the reaction product for GC–MS detection. Lineweaver–Burk plots were constructed to obtain the Km value and Kcat. All assays were conducted in triplicate.

The GC–MS system used was an Agilent 7890a gas chromatograph/5975c mass selective detector with a 30-m DB-5MS capillary column. The carrier gas was supplied at a constant flow rate of 1 mL/min. The oven conditions were as follows: initial temperature of 40 °C for 2 min, increased by 10 °C/min to 130 °C, held for 5 min, increased by 8 °C/min to 230 °C, held for 3 min. The inlet temperature was kept constant at 250 °C, and the MS transfer line was set at 290 °C. The MS acquisition parameters included scanning from 50 to 550 m/z in the electron impact (EI) mode for routine analysis.

## Supplementary Information

**Additional file 1: Fig. S1.** Morphology of *M. paniculata* tree and flowers at different developmental stages. **Fig. S2.** The 21-mer distribution generated by *M. paniculata* whole genome NGS reads. **Fig. S3.** Hi-C contact heatmap of *M. paniculata*. **Fig. S4.** Comparison of Ks distributions of inter- and intra-species homologous gene pairs for *M. paniculata* and *C. sinensis*. **Fig. S5.** Collinearity analysis between *M. paniculata* genome and those of *C. sinensis*, *C. maxima,* and *P. trifoliata*. **Fig. S6.** Repeat lengths in different regions of the genomes. **Fig. S7.** Numbers and percentages of different TE types in genomes of four Rutaceae species. **Fig. S8.** Gene percentages with different types of TEs inserting in 10-kb upstream and downstream regions in four Rutaceae genomes. **Fig. S9.** Phylogenetic trees of Copia and Gypsy transposase in four Rutaceae genomes. **Fig. S10.** Collinearity among *PAAS* gene regions in *P. trifoliata*, *M. paniculata*, *C. maxima*, and *C. sinensis.* **Fig. S11.** Detailed schematic diagram of the structure of the *PAAS* gene regions in *C. sinensis*. **Fig. S12.** Enzymatic characterization of PAASs.

**Additional file 2: Table S1.** Nanopore, Illumina, Hi-C, sequencing data for *M. paniculata*. **Table S2.** Chromosome length statistics. **Table S3.** Summary of transposable elements in the genomes of *M. paniculate*, *P. trifoliata*, *C. maxima*, *C. sinensis*. **Table S4.** Functional annotations of genes in *M. paniculata.* **Table S5.** miRNA statistics. **Table S6.** Genome BUSCO results of the *M. paniculate* genome assembly. **Table S7.** Protein BUSCO results of the *M. paniculate* genome assembly. **Table S8.** Repeat sequence statistics among different genome regions. **Table S9.** Repeat sequence statistics in intron. **Table S10.** The optimised PAAS gene sequences according to the *E. coli* codon. **Table S11.** Summary of PAAS steady-state kinetic data with L-Phe as substrate. **Table S12.** The primers for pET28a vector construction.

**Additional file 3.** KEGG pathway enrichment analysis of genes in expanded gene families of *M. paniculata*.

**Additional file 4.** KEGG pathway enrichment analysis of genes with LTR insertions in the 3-kb upstream region in *M. paniculata*.

**Additional file 5.** Volatiles in three flowering stages of *M. paniculata*.

**Additional file 6.** Volatiles in three flowering stages of *C. maxima*.

**Additional file 7.** TPMs of genes in three flowering stages, leaves, and stemswith three replicates.

**Additional file 8.** Production of phenylacetaldehyde catalyzed by *M. paniculata PAAS* genes with three replicates.

## Data Availability

All data generated or analysed during this study are included in this published article, its supplementary information files and publicly available repositories. The datasets generated during the current study are available in the National Genomics Data Canter [68]. Data archives for *M. paniculata* genome assembly and annotation have been deposited under BioProject PRJCA009823 with the accession number of CRA007517 for raw sequencing data and GWHBPAQ00000000 for genome FASTA and annotation GFF3 files [69]. RNA-seq data for *M. paniculata* and *C. maxima* have been deposited under BioProject PRJCA010771 with assession number of CRA007603 [70] and BioProject PRJCA009845 with assession number of CRA007077 [71], respectively.

## Abbreviations

PAAS: Phenylacetaldehyde synthase
LTR: Long terminal repeat
MYA: Million years ago
WGD: Whole genome duplication
TEs: Transposable elements
TIR: Terminal inverted repeat
HS-SPME: Headspace solid-phase microextraction
GC-MS: Gas chromatography-mass spectrometry
PCA: Principal component analysis
EDTA: Extensive de novo TE Annotator
BUSCO: Benchmarking Universal Single-Copy Orthologs
Ks: Synonymous nucleotide substitutions
PLP: Pyridoxal 5-phosphate
EI: Electron impact
