# Supplementary material for: Chromosome-level genome assembly of Murraya paniculata sheds light on biosynthesis of floral volatiles
Source: BMC Biol. 2023 Jun 20;21:142. doi: 10.1186/s12915-023-01639-6 (PMC10283294; doi:10.1186/s12915-023-01639-6)
Supplement: Supplementary file 1 — Additional file 1: Fig. S1. Morphology of M. paniculata tree and flowers at different developmental stages. Fig. S2. The 21-mer distribution generated by M. paniculata whole genome NGS reads. Fig. S3. Hi-C contact heatmap of M. paniculata. Fig. S4. Comparison of Ks distributions of inter- and intra-species homologous gene pairs for M. paniculata and C. sinensis. Fig. S5. Collinearity analysis between M. paniculata genome and those of C. sinensis, C. maxima, and P. trifoliata. Fig. S6. Repeat lengths in different regions of the genomes. Fig. S7. Numbers and percentages of different TE types in genomes of four Rutaceae species. Fig. S8. Gene percentages with different types of TEs inserting in 10-kb upstream and downstream regions in four Rutaceae genomes. Fig. S9. Phylogenetic trees of Copia and Gypsy transposase in four Rutaceae genomes. Fig. S10. Collinearity among PAAS gene regions in P. trifoliata, M. paniculata, C. maxima, and C. sinensis. Fig. S11. Detailed schematic diagram of the structure of the PAAS gene regions in C. sinensis. Fig. S12. Enzymatic characterization of PAASs. [file 12915_2023_1639_MOESM1_ESM.docx]

Fig. S1. Morphology of *M. paniculata* tree and flowers at different developmental stages.


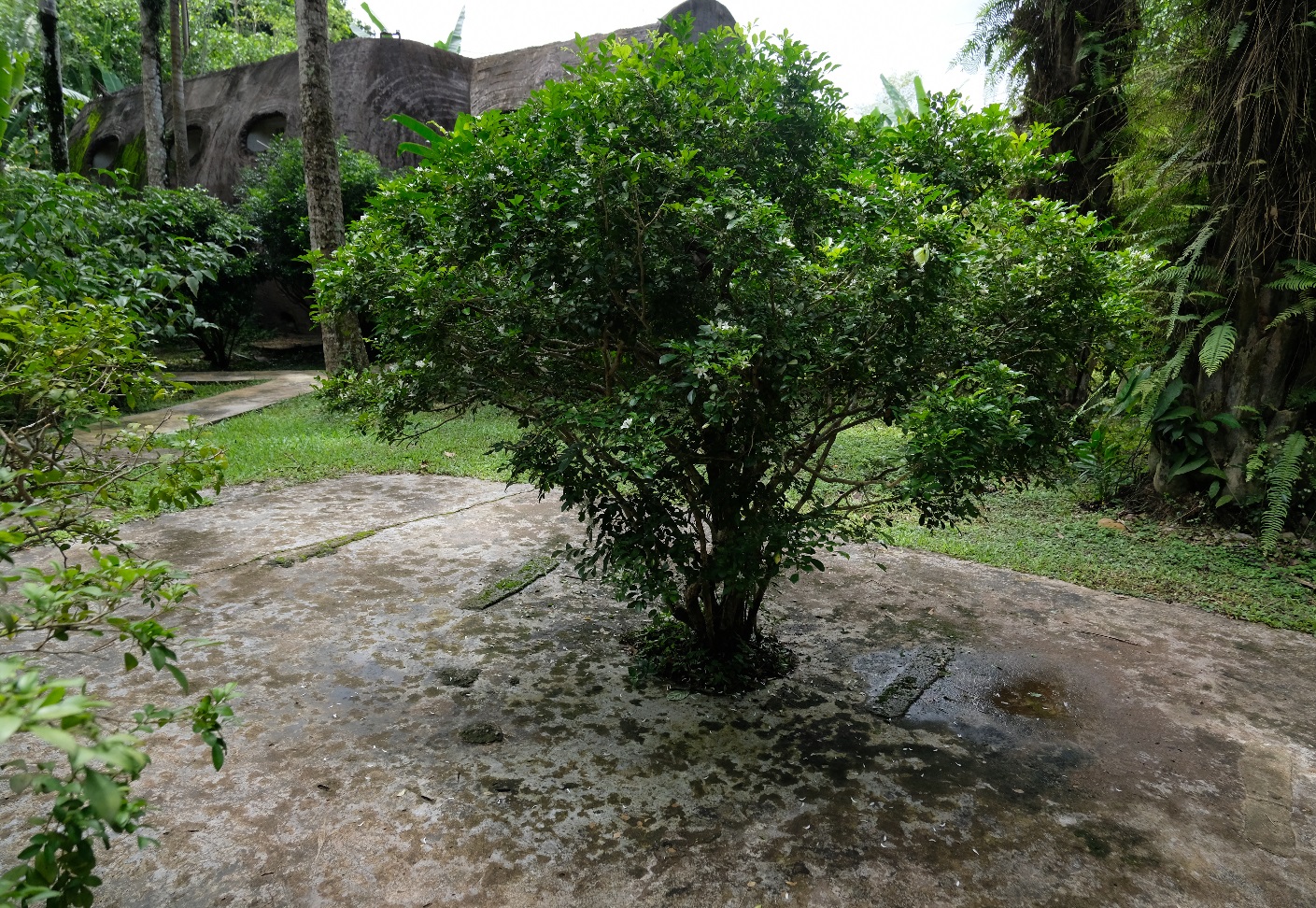

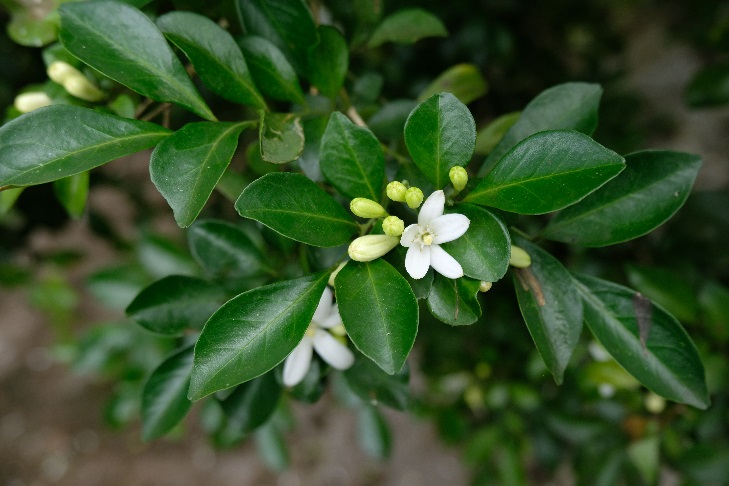


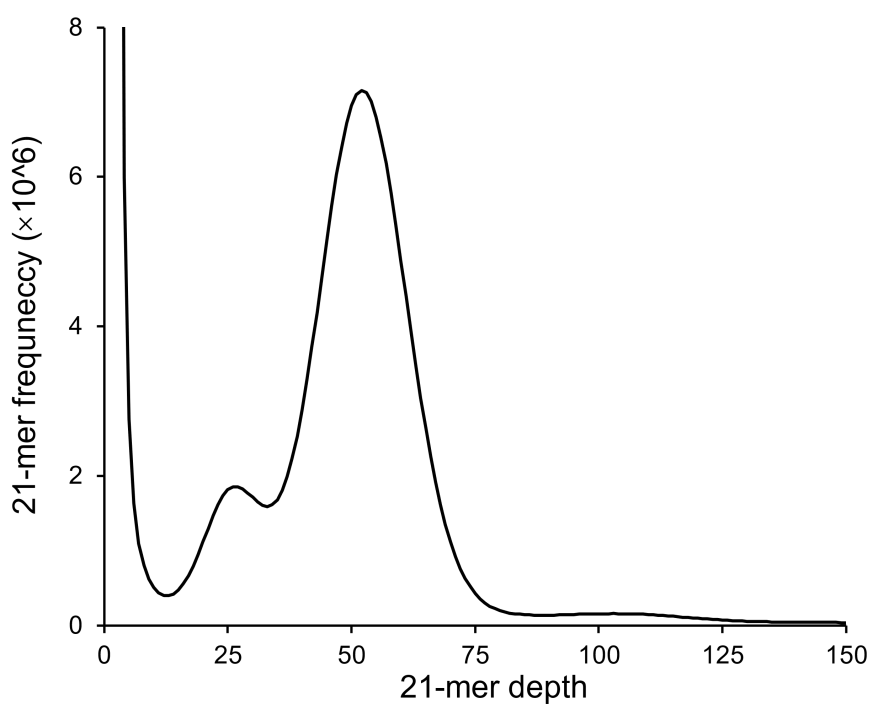
Fig. S2. The 21-mer distribution generated by *M. paniculata* whole genome NGS reads.

Fig. S3. Hi-C contact heatmap of *M. paniculata*. Chr1 to Chr9 represent the nine chromosomes inferred by 3D-DNA. Light pink to dark red colour indicates frequency of Hi-C interactive links from low to high.
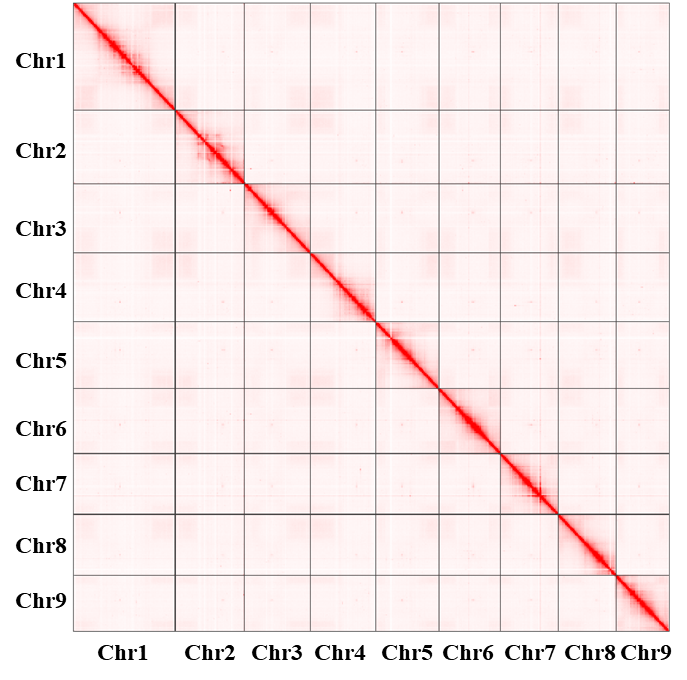


Fig. S4.
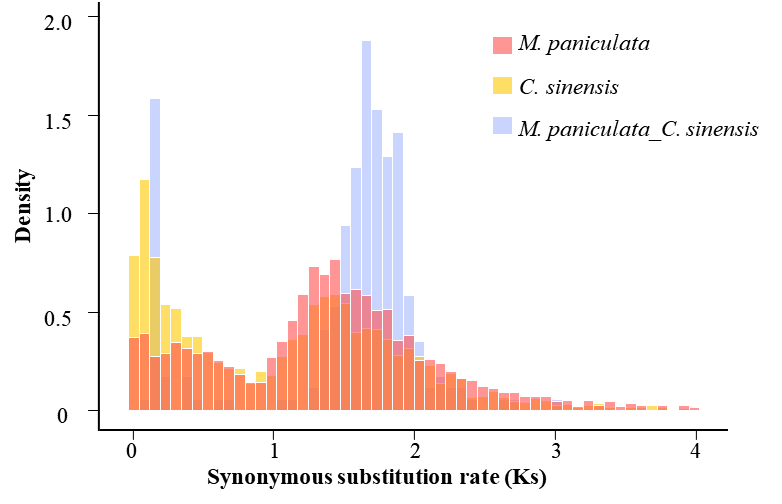
 Comparison of Ks distributions of inter- and intra-species homologous gene pairs for *M. paniculata* and *C. sinensis*.


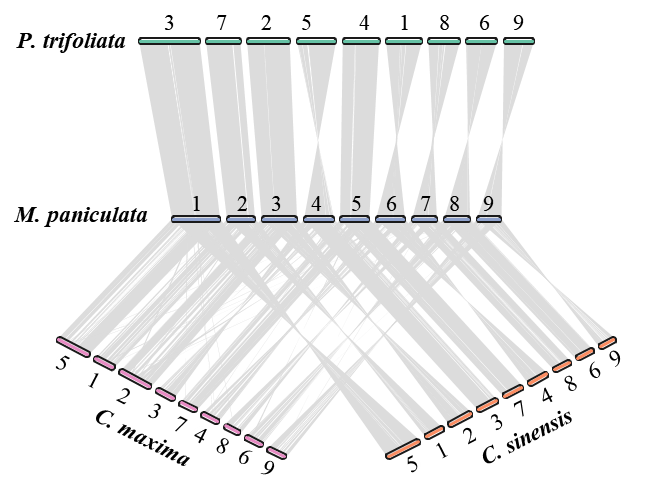
Fig. S5. Collinearity analysis between *M. paniculata* genome and those of *C. sinensis*, *C. maxima,* and *P. trifoliata*. Numbers represent the chromosome numbers, and grey lines represent all collinearity blocks between the genomes.


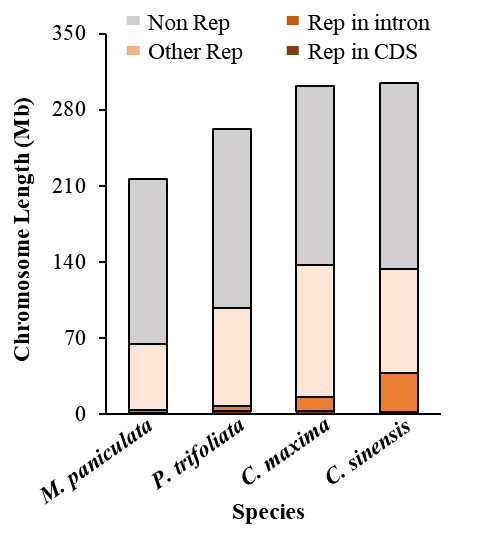
Fig. S6. Repeat lengths in different regions of the genomes. Rep: repeat sequences.


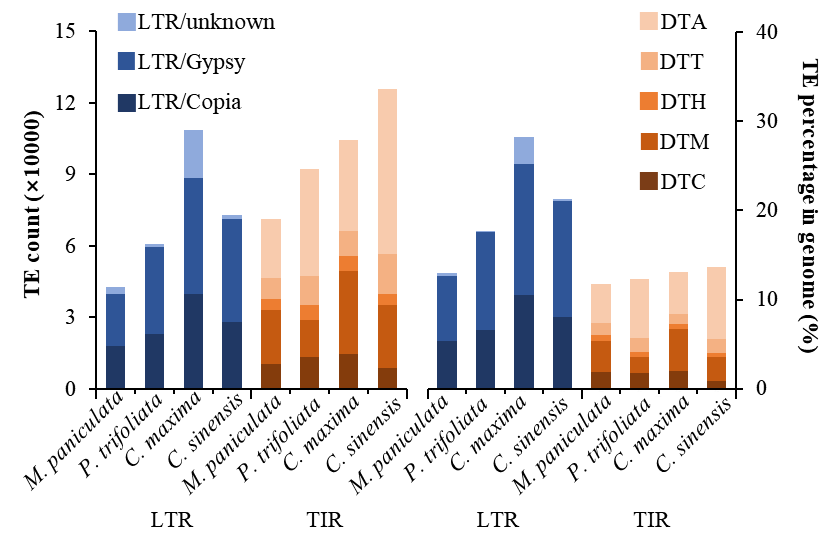
Fig. S7. Numbers and percentages of different TE types in genomes of four Rutaceae species. Long terminal repeat, LTR; terminal inverted repeats, TIR. Classifications of TIRs: Tc1-Mariner, DTT; hAT, DTA; Mutator, DTM; PIF-Harbinger, DTH; CACAT, DTC.

Fig. S8. Gene percentages with different types of TEs inserting in 10-kb upstream and downstream regions in four Rutaceae genomes.
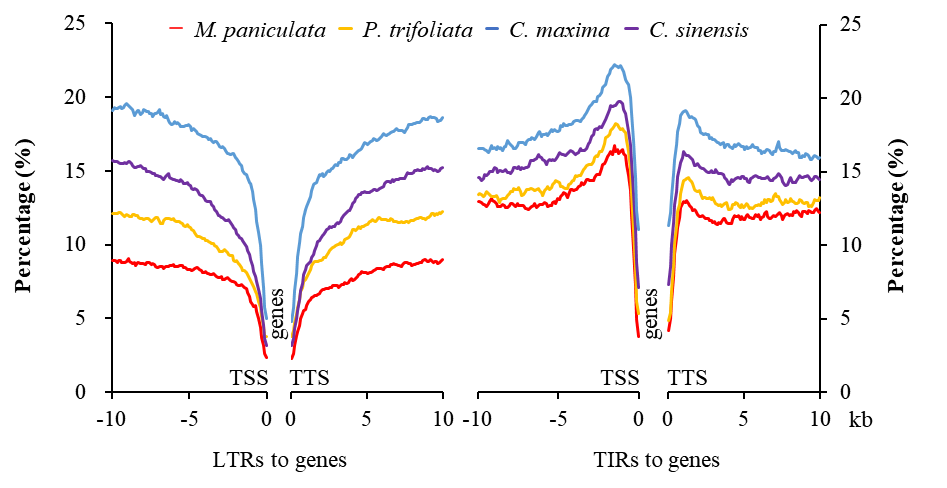
 Curves were generated from 100-bp sliding windows.

Fig. S9.
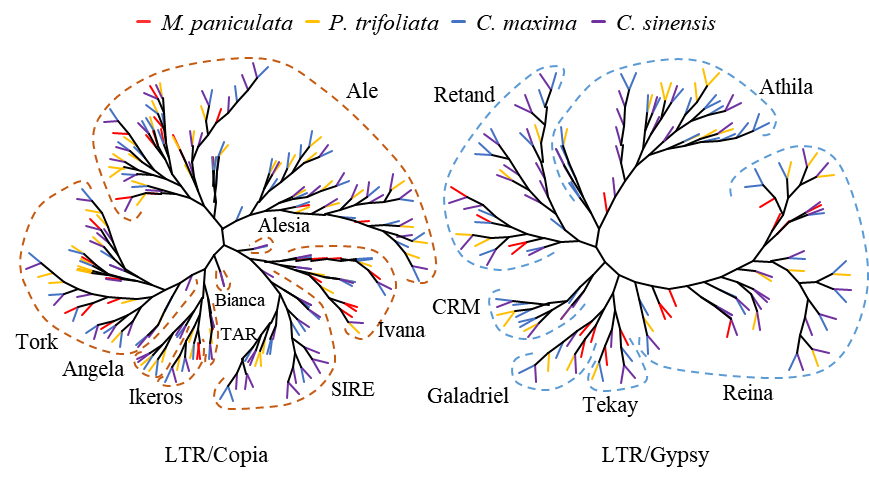
 Phylogenetic trees of Copia and Gypsy transposase in four Rutaceae genomes. Sequences were aligned with MAFFT based on conserved transposase protein sequences. Trees were generated using IQTREE2 with the JTTDCMut+F+R6 model.


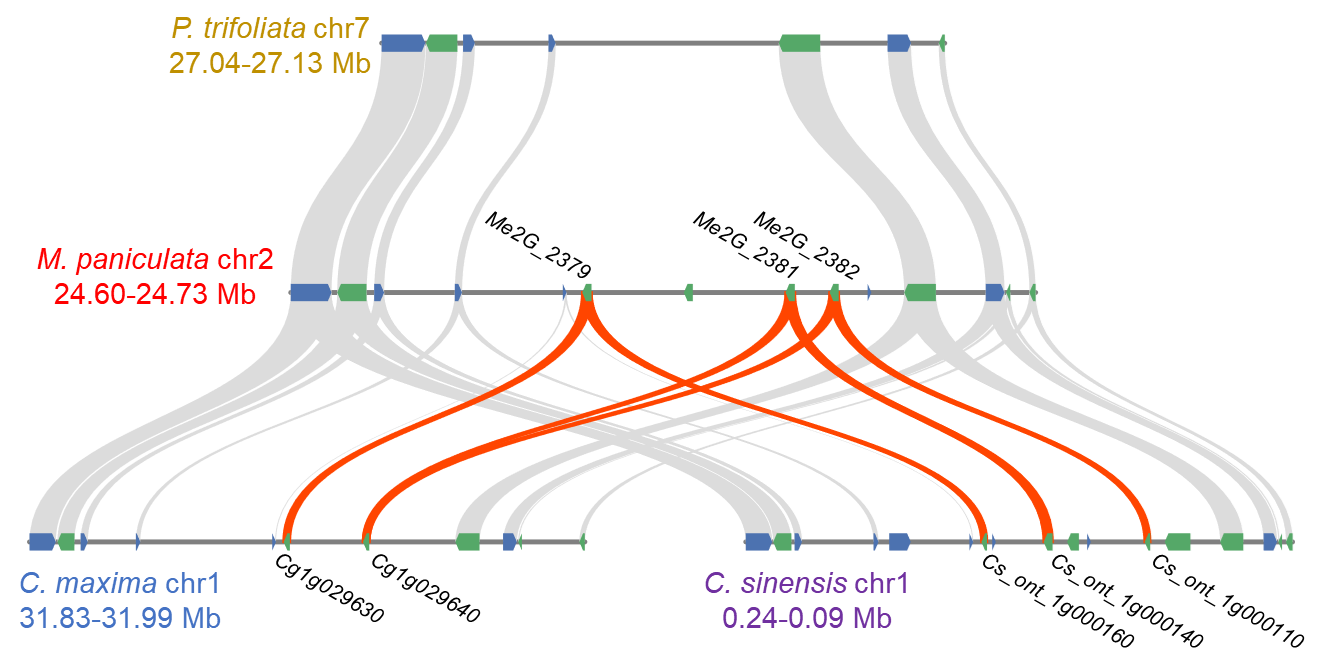
Fig. S10. Collinearity among *PAAS* gene regions in *P. trifoliata*, *M. paniculata*, *C. maxima*, and *C. sinensis.*


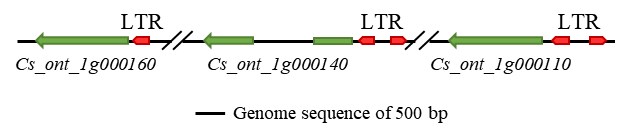
Fig. S11. Detailed schematic diagram of the structure of the *PAAS* gene regions in *C. sinensis*. Black line represents genome sequence; green bold arrow represents length and orientation of CDS; red bold arrow represents length and orientation of LTR fragment.


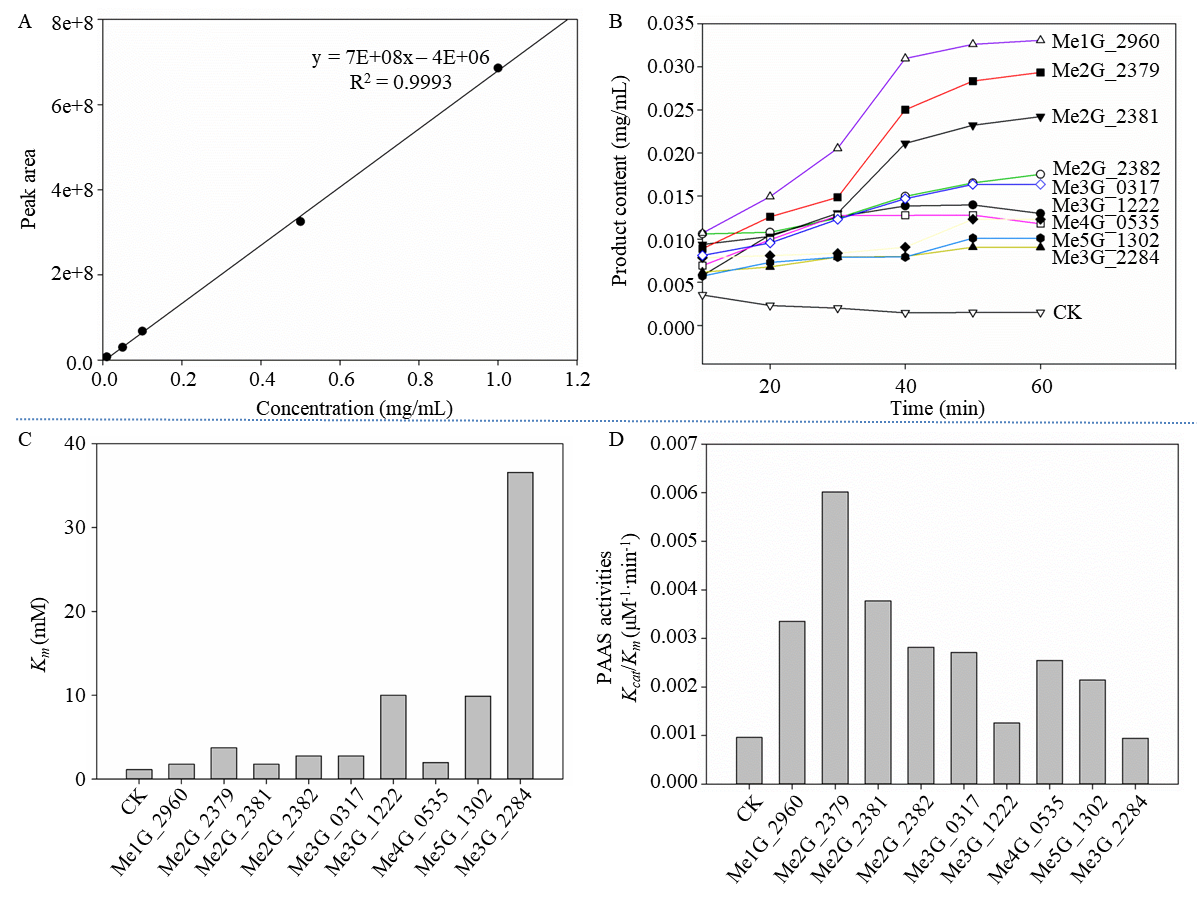
Fig. S12. Enzymatic characterization of PAASs. *In vitro* enzyme activity assays of CK and 9 PAASs with phenylalanine as the substrate show the corresponding catalytic efficiency (Kcat/Km) values. Dependence of PAAS activity on time and substrate, calculated from peak area ratios of phenylacetaldehyde to the standard curve. Lineweaver-Burk plots were constructed to obtain the Km and Kcat values. **A.** Standard curve of phenylacetaldehyde. **B.** Product generation time curve. **C.** The concentration of substrate required for the PAAS enzyme to carry out the reaction. **D.** Lineweaver-Burk plot of PAAS. All assays were conducted in triplicate.
