# Supplementary material for: Chromosome-level genome assembly of Murraya paniculata sheds light on biosynthesis of floral volatiles
Source: BMC Biol. 2023 Jun 20;21:142. doi: 10.1186/s12915-023-01639-6 (PMC10283294; doi:10.1186/s12915-023-01639-6)
Supplement: Supplementary file 2 — Additional file 2: Table S1. Nanopore, Illumina, Hi-C, sequencing data for M. paniculata. Table S2. Chromosome length statistics. Table S3. Summary of transposable elements in the genomes of M. paniculate, P. trifoliata, C. maxima, C. sinensis. Table S4. Functional annotations of genes in M. paniculata. Table S5. miRNA statistics. Table S6. Genome BUSCO results of the M. paniculate genome assembly. Table S7. Protein BUSCO results of the M. paniculate genome assembly. Table S8. Repeat sequence statistics among different genome regions. Table S9. Repeat sequence statistics in intron. Table S10. The optimised PAAS gene sequences according to the E. coli codon. Table S11. Summary of PAAS steady-state kinetic data with L-Phe as substrate. Table S12. The primers for pET28a vector construction. [file 12915_2023_1639_MOESM2_ESM.docx]

**Table S1.** Nanopore, Illumina, Hi-C, sequencing data for the *M. paniculata*.

| **Nanopore sequencing data** | | | | |
| --- | --- | --- | --- | --- |
| Raw Bases (bp) | Raw Reads Count | Coverage Depth | Reads N50 (bp) | Raw Bases (bp) |
| 34136583499 | 1702577 | 142X | 27386 | 34136583499 |
| **Illumina sequencing data** | | | | |
| Raw Bases (bp) | Raw Reads Count | Clean Bases (bp) | Q20 (%) | Q30 (%) |
| 18849697800 | 125664652 | 18574959974 | 72.56 | 97.29% |
| **Hic sequencing data** | | | | |
| Raw Bases (bp) | Raw Reads Count | Clean Bases (bp) | Clean Reads Q20 | Clean Reads Q30 |
| 45180090900 | 301200606 | 41785092476 | 96.62% | 89.68% |

**Table S2.** Chromosome length statistics.

| **Pseudomolecules** | **Length (bp)** | **Gap number** |
| --- | --- | --- |
| chromosome1 | 36719023 | 3 |
| chromosome2 | 25111180 | 8 |
| chromosome3 | 24135235 | 9 |
| chromosome4 | 23920500 | 9 |
| chromosome5 | 22745385 | 2 |
| chromosome6 | 22503019 | 1 |
| chromosome7 | 20966149 | 6 |
| chromosome8 | 20672466 | 4 |
| chromosome9 | 19541161 | 3 |

**Table S3.** Summary of transposable elements in the genomes of *M. paniculate*, *P. trifoliata*, *C. maxima*, *C. sinensis*.

| ***M. paniculata*** | | | | | |
| --- | --- | --- | --- | --- | --- |
|  | Class | subclass | Count | bpMasked | % masked |
| retrotransposon | LTR |  | | | |
|  |  | Copia | 17960 | 11648514 | 5.37% |
|  |  | Gypsy | 21978 | 15812768 | 7.29% |
|  |  | unknown | 2746 | 777416 | 0.36% |
|  | nonLTR |  | | | |
|  |  | L1_LINE | 790 | 285573 | 0.13% |
|  |  | LINE_element | 431 | 195633 | 0.09% |
|  |  | Penelope | 127 | 67939 | 0.03% |
|  |  | tRNA_SINE | 563 | 114045 | 0.05% |
|  |  | others | 5025 | 1935949 | 0.90% |
| transposon | TIR |  | | | |
|  |  | CACTA | 10598 | 4062475 | 1.87% |
|  |  | Mutator | 22432 | 7590386 | 3.50% |
|  |  | P | 34 | 55099 | 0.03% |
|  |  | PIF_Harbinger | 4834 | 1492628 | 0.69% |
|  |  | Tc1_Mariner | 8492 | 2760839 | 1.27% |
|  |  | hAT | 24876 | 9570588 | 4.41% |
|  |  | others | 7888 | 2495579 | 1.15% |
|  | nonTIR |  | | | |
|  |  | helitron | 14787 | 5142946 | 2.37% |
| low_complexity |  | | 55 | 50217 | 0.02% |
| Total |  | | 143616 | 64058594 | 29.54% |
| ***P. trifoliata*** | | | | | |
|  | Class | subclass | Count | bpMasked | %masked |
| retrotransposon | LTR |  | | | |
|  |  | Copia | 23092 | 17278967 | 6.54% |
|  |  | Gypsy | 36492 | 29226786 | 11.06% |
|  |  | unknown | 1039 | 313060 | 0.12% |
|  | nonLTR |  | | | |
|  |  | I_LINE | 39 | 28456 | 0.01% |
|  |  | L1_LINE | 2914 | 3091022 | 1.17% |
|  |  | Penelope | 516 | 163251 | 0.06% |
|  |  | tRNA_SINE | 170 | 21907 | 0.01% |
|  |  | others | 16916 | 7885028 | 2.98% |
| transposon | TIR |  | | | |
|  |  | CACTA | 13501 | 4634494 | 1.75% |
|  |  | Mutator | 15450 | 4676359 | 1.77% |
|  |  | P | 78 | 16368 | 0.01% |
|  |  | PIF_Harbinger | 6402 | 1646739 | 0.62% |
|  |  | Tc1_Mariner | 11969 | 4087624 | 1.55% |
|  |  | hAT | 44735 | 17501094 | 6.62% |
|  |  | others | 26311 | 8282938 | 3.13% |
|  | nonTIR |  | | | |
|  |  | helitron | 121 | 89362 | 0.03% |
| low_complexity |  | | 16 | 50026 | 0.02% |
| Total |  | | 199761 | 98993481 | 37.45% |
| ***C. maxima*** |  | | | | |
|  | Class | subclass | Count | bpMasked | %masked |
| retrotransposon | LTR |  | | | |
|  |  | Copia | 39899 | 31653587 | 10.50% |
|  |  | Gypsy | 48773 | 44198529 | 14.66% |
|  |  | unknown | 20066 | 9311518 | 3.09% |
|  | nonLTR |  | | | |
|  |  | DIRS_YR | 42 | 10048 | 0.00% |
|  |  | I_LINE | 68 | 20859 | 0.01% |
|  |  | L1_LINE | 433 | 138321 | 0.05% |
|  |  | LINE_element | 1838 | 1004842 | 0.33% |
|  |  | Penelope | 809 | 244709 | 0.08% |
|  |  | tRNA_SINE | 159 | 19138 | 0.01% |
|  |  | others | 5977 | 2179508 | 0.72% |
| transposon | TIR |  | | | |
|  |  | CACTA | 14634 | 6097629 | 2.02% |
|  |  | Mutator | 34650 | 13967856 | 4.63% |
|  |  | PIF_Harbinger | 6516 | 1889907 | 0.63% |
|  |  | Tc1_Mariner | 10243 | 3439700 | 1.14% |
|  |  | hAT | 38445 | 14091155 | 4.68% |
|  |  | others | 14435 | 4336211 | 1.44% |
|  | nonTIR |  | | | |
|  |  | helitron | 15229 | 4148105 | 1.38% |
| low_complexity |  | | 21 | 64859 | 0.02% |
| Total |  | | 252237 | 1.37E+08 | 45.39% |
| ***C. sinensis*** | | | | | |
|  | Class | subclass | Count | bpMasked | %masked |
| retrotransposon | LTR |  | | | |
|  |  | Copia | 28147 | 24644792 | 8.10% |
|  |  | Gypsy | 43228 | 39619636 | 13.02% |
|  |  | unknown | 1435 | 608053 | 0.20% |
|  | nonLTR |  | | | |
|  |  | DIRS_YR | 54 | 10583 | 0.00% |
|  |  | I_LINE | 75 | 23875 | 0.01% |
|  |  | L1_LINE | 3780 | 1793650 | 0.59% |
|  |  | Penelope | 2614 | 796600 | 0.26% |
|  |  | tRNA_SINE | 132 | 17611 | 0.01% |
|  |  | others | 27848 | 10602670 | 3.49% |
| transposon | TIR |  | | | |
|  |  | CACTA | 8617 | 2514982 | 0.83% |
|  |  | Mutator | 26406 | 8157910 | 2.68% |
|  |  | PIF_Harbinger | 4988 | 1503258 | 0.49% |
|  |  | Tc1_Mariner | 16694 | 4771332 | 1.57% |
|  |  | hAT | 69262 | 24606101 | 8.09% |
|  |  | others | 40383 | 13635341 | 4.48% |
|  | nonTIR |  | | | |
|  |  | helitron | 420 | 142281 | 0.05% |
| low_complexity |  | | 29 | 64636 | 0.02% |
| total |  | | 274112 | 1.34E+08 | 43.89% |

**Table S4.** Functional annotations of genes in *M. paniculate.*

| **Function annotation** | **Count** | **Percentage** |
| --- | --- | --- |
| total proteins | 23548 | 100.00% |
| GO | 10709 | 45.48% |
| KEGG | 10440 | 44.33% |
| Swiss-Prot | 16703 | 70.93% |
| eggNOG_OGs | 22201 | 94.28% |
| NR | 22647 | 96.17% |

**Table S5.** miRNA statistics.

| Non-coding RNA | Count |
| --- | --- |
| miRNA | 163 |
| rRNA | 246 |
| tRNA | 481 |

**Table S6.** Genome BUSCO results of the *M. paniculate* genome assembly.

| **Genome BUSCO** | **Complete BUSCOs** | **Complete and single-copy BUSCOs** | **Complete and duplicated BUSCOs** | **Fragmented BUSCOs** | **Missing BUSCOs** | **Total BUSCO groups searched** |
| --- | --- | --- | --- | --- | --- | --- |
| Gene number | 2285 | 2259 | 26 | 9 | 32 | 2326 |
| Percentage | 98.24% | 97.12% | 1.12% | 0.39% | 1.38% | 100.00% |

**Table S7.** Protein BUSCO results of the *M. paniculate* genome assembly.

| **Protein BUSCO** | **Complete BUSCOs** | **Complete and single-copy BUSCOs** | **Complete and duplicated BUSCOs** | **Fragmented BUSCOs** | **Missing BUSCOs** | **Total BUSCO groups searched** |
| --- | --- | --- | --- | --- | --- | --- |
| Protein number | 2290 | 2274 | 16 | 5 | 31 | 2326 |
| Percentage | 98.45% | 97.76% | 0.69% | 0.21% | 1.33% | 100.00% |

**Table S8.** Repeat sequence statistics among different genome regions.

| **Repeat length (bp)** | ***M. paniculata*** | ***P. trifoliata*** | ***C. maxima*** | ***C. sinensis*** |
| --- | --- | --- | --- | --- |
| Repeat in CDS | 1380236 | 3029106 | 2536929 | 1693957 |
| Repeat in intron | 2071929 | 4341319 | 12963934 | 35943603 |
| Other Repeat | 60879745 | 89905676 | 121629034 | 96222400 |
| Non Repeat | 151982208 | 165254344 | 164826677 | 170356259 |
| Total Repeat | 64331910 | 97276101 | 137129897 | 133859960 |

**Table S9.** Repeat sequence statistics in intron.

|  | ***M. paniculata*** | ***P. trifoliata*** | ***C. maxima*** | ***C. sinensis*** |
| --- | --- | --- | --- | --- |
| Percentage of genes with TE in intron | 21.24% | 23.46% | 26.85% | 28.34% |
| Number of ClassI TEs per intron | 0.023497 | 0.039348 | 0.0944 | 0.093946 |
| Number of ClassII TEs per intron | 0.085059 | 0.09007 | 0.174743 | 0.195014 |

**Table S10.** The optimized PAAS gene sequences according to the *E. coli* codon.

| **>Me1G_2960**  ATGGATGCAGAGCAGCTGCGTGAAAACGCTCATAAAATGGTAGATTTCATTGCGGACTACTACAAATCTATCGAAAACTTTCCAGTTCTGAGCCAAGTACAGCCGGGTTATCTGCACAACCTGATCCCGGATTCTGCTCCTAACCATCCAGAATCCCTGCAGAACGTTCTGGATGACATCCAGGAAAAAATTCTGCCGGGTGTCACCCACTGGCAGTCTCCGAACTATTTTGCATACTTCCCGTCTAACTCCTCCGTTGCCGGTTTCCTGGGTGAAATGCTGAGCGCAGGTCTGAACATCGTGGGTTTCTCTTGGATCACGTCTCCTGCGGCCACCGAACTGGAAATGATTGTGCTGGATTGGCTGGCCAAACTGCTGAAACTGCCGGAAGATTTTCTGAGCACCGGCCAGGGTGGTGGTGTAATTCAGGGCACGGCATCTGAGGCAGTACTGGTTGTTCTGCTGGCAGCCCGTGATGAAGCGCTGAAACGCGTCGGTAAAAACTCCCTGGAAAAACTGGTTGTCTACGCATCCGATCAAACTCACTCCGCGCTGCAAAAAGCGTGCCAGATTGGTGGCATTCATCGTCAGAACTTCCGCGTTCTGAAAACCGATTCTAGCACTAACTATAGCCTGTCCCCTGATTCTCTGGCCGAGGCGATCAGCCGTGATCTGACGATGGGCCTGATCCCTTTCTTTCTGGGCGCAACTGTTGGTACCACCTCTTCTACTGCAGTAGACCCGCTGCTGGCTCTGGGTAACATTGCAAAAAGCAACGGTATGTGGTTCCACGTTGATGCCGCGTACGCGGGCAGCGCATGCATTTGCCCGGAATACCGCCAGTACATCGACGGTGTGGAAAAAGCCGACAGCTTCAACATGAACGCGCACAAATGGTTCCTGACTAATTTCGACTGCAGCGCACTGTGGGTGAAGGATCGTAACACCCTGATTCAATCTCTGAGCACCAACCCGGAATTCCTGAAAAACAAGGTCAGCTTTTACTTCTCCGTTCACAACATGTTCCTGTACTACTTCCTGGCACAGCTGTCTCGTCCGAAATCCAAATCCCACCTGTTCGTCTTCGTGCATATTAACTGCATCATGAACTGCCCGTTCAACAACGTTATGCTGACCGTTGTTGAACTGTCTACCCTGAGCGGTGAGAACGGTAACATTCTGGCCTCCCAGGCTAACATGGTTGTGGACTACAAAGATTGGCAGATCCCACTGGGCCGTCGCTTCCGTTCTCTGAAACTGTGGATGGTACTGCGCCTGTATGGTCTGGAAAACCTGCAGGGCTACATCCGCAACCACATTCAGCTGGCAAAACACTTTGAAGGTCTGGTTGCTCAGGATTCTCGTTTCGAAGTAGTAACCCCGCGCATTTTCTCTCTGGTTTGCTTCCGTCTGCTGCCGCCGCACAACGATGAGGACCACGGTAACAAACTGAACCATAACCTGCTGGACGCCGTCAACTCTACTGGCAAAATCTTCATTAGCCATACTGTTCTGTCCGGTAAATACATCCTGCGCTTCGCCGTTGGTGCGCCTCTGACTGAAGGTCGTCACGTAAACGCTGCCTGGGAGGTGGTCCGTGATCGTGCCAGCGCGCTGCTGGCACGCCTGTCTACCGAA  **>Me2G_2379**  ATGGGCTCTTTTGCACTGCCTCCGAACGGTAACACCCACGGCCGCTCCTTCTCTGCGGATCTGGAACCGAAGTCCTTCGCAGACGAAGCGAAAGCCGTGATCGATTTCATCGCAGATTACTACAAAAACATTGAGAAGTATCCTGTGCAGAGCAAAGTTGAACCGGGTTACCTGTCCGCGCAGCTGCCTGATACTGCACCGCACTCTCCGGAGTCTCTGGACGACATCCTGAAAGACGTCACCGATTCCATCATTCCGGGTCTGACTCATTGGCAATCTCCGAACTTCTTCGGTTATTTTCAGGCGAATGCTTCTACTGCAGGCTTTCTGGGTGAGATGCTGTGCAGCGGTTTCAACGTAGTAGGTTTCAACTGGCTGGCATCTCCGGCAGCAACTGAACTGGAATCCATCGTTATGGACTGGATGGGTAAAATGCTGAAGCTGCCGTCTTCTTTCCTGTTCTCCGGTACCGGTGGTGGTGTTCTGCACGGCTCCACTTGCGAATCTCTGGTTTGCACCCTGGCTGCTGCCCGTGATGAAGTGCTGGAAAAACTGGGTGGCGGCTTTGATAACATCACCAAACTGGTGGTTTACGCGAGCGACCAGACCCACTTCGCACTGCAGAAATCCGCTAAACTGATCGGTATCCCTCCGGCAAATTTCCGTCCGCTGTCCACCTCCTTTTCCACCGAGTTCAGCCTGTCCCCGGATGCGGTTCGTGCAGCGATCGAGGATGATATCAAATCTGGCTACGTTCCTCTGTACCTGTGTGCCACCGTTGGTACCACTGGCGCAGGTGCCGTGGACCCGATCGAAGAACTGGGTAAAATCGCAACCGAGTATAAGCTGTGGCTGCATATCGATGCTGCATATGCAGGTGGTGCTTGTATCTGCCCGGAATATCGTCACTATCTGAACGGTGTGGAACTGGCTGATTCTATCTCCCTGAACCCACACAAATGGTTCCTGACCAATATGGACTGTGGCTGTCTGTGGGTTAAACACCCGTCTTTTCTGGTTGGCTCTCTGTCTACGAAATCTGATATCATGCGCAGCCGTTCCCCTGCGTCTTCCACCTCTGCAAACGCTGCCCCAGTAATTGACTACAAAGATTGGCAGATCGCTCTGTCTCGTCGTTTCAAAGCGCTGAAGCTGTGGACCGTTATCCGTAAACACGGCTACTCTGGCCTGACTTATCATATCCGTTCCGATGTTAACATGGCGAAACGTTTCGAGGCTATGGTGGCGAAAGATGAACGTTTCGAGATTGCGGTACCGCGCAAATTCGCACTGGTTTGTTTCCGTCTGAAACCGAAACGTGAAAGCGAAGGCACGGAACTGAACCGTAAACTGCTGGACGCGCTGAACGCGTCCGGCCGTGCATTCCTGACTCAGGCTGTGCTGGGTGGTGTGTATGTGATCCGTTGCAGCATCGGTACGACGCTGACCCAAGATTCTCATGTTGATGACCTGTGGAAACTGGTGCAGGAAAAAGCGGACCGCCTGCTGAGCCTGCAGGAACCGGAACACGCACGTTGT  **>Me2G_2381**  ATGGGTTCTTTTGCTCTGCCTGCCAACGCCAATACTCATGGTGGTTCTTTTTCCGCTGACCTGGAACCGAAATCCTTTGCGGACGAGGCCAAAGCAGTGATCGACTTCATCGCGGACTATTATAAAAATATCGAGAAGTACCCGGTGCAGAGCAAAGTAGAGCCTGGTTATCTGTCCGCACAACTGCCGGATACTGCTCCGCATAGCCCGGAGTCCCTGGACGACATTCTGAAAGATGTTACTGACTCCATTATCCCGGGTCTGACCCACTGGCAATCTCCGAACTTTTTCGGTTACTTTCAGGCTAACGCGTCTACCGCTGGTTTCCTGGGTGAAATGCTGTGTTCCGGCTTCAACGTGGTGGGTTTCAATTGGCTGGCTTCTCCGGCAGCAACTGAACTGGAATCTATCGTTATGGACTGGATGGGTAAAATGCTGAAACTGCCGTCCTCCTTTCTGTTTTCTGGTACCGGTGGCGGCGTTCTGCACGGTAGCACCTGTGAATCTCTGGTGTGCACCCTGGCTGCGGCTCGTGATGAAGTGCTGGAAAAGCTGGGCGGTGGTTTTGACAACATCACCAAGCTGGTTGTGTACGCGAGCGATCAGACCCACTTCGCACTGCAGAAAAGCGCGAAACTGATCGGCATCCCGCCGGCTAACTTTCGTCCGCTGTCCACTTCCTTCTCTACCGAATTCAGCCTGTCCCCTGATGCAGTGCGTGCCGCGATCGAAGACGACATCAAAAGCGGTTACGTTCCGCTGTACCTGTGCGCAACCGTTGGCACCACTGGTGCGGGCGCAGTTGATCCGATTGAAGAACTGGGCAAGATTGCGACCGAATACAAACTGTGGCTGCACATCGACGCGGCGTATGCCGGTGGCGCATGTATTTGTCCGGAATACCGCCACTACCTGAACGGCGTTGAGCTGGCTGATAGCATCTCTCTGAACCCGCACAAATGGTTCCTGACCAACATGGATTGTGGCTGCCTGTGGGTTAAACACCCGAGCTTCCTGGTTGGCTCCCTGTCCACGAAGTCCGATATCATGCGTTCTCGTTCTCCAGCGAGCAGCACTCGTGCGAATGCGGCGCCGGTTATCGACTACAAAGATTGGCAGATTGCACTGTCTCGTCGCTTCAAAGCTCTGAAACTGTGGACTGTCATCCGCAAACACGGTTACAGCGGCCTGACTTATCACATCCGTTCTGACGTCAACATGGCGAAACGTTTCGAAGCAATGGTTGCTAAAGACGAACGTTTCGAAATCGCAGTGCCGCGTAAGTTTGCTCTGGTTTGCTTCCGCCTGAAACCAAAACGTGAATCCGAAGGCACGGAACTGAACCGCAAACTGCTGGATGCTCTGAACGCTTCTGGTCGTGCGTTTCTGACCCAGGCAGTTCTGGGTGGCGTGTACGTTATCCGTTGCTCCATCGGCACCACCCTGACCCAGGACTCTCACGTTGATGACCTGTGGAAACTGGTACAAGAAAAAGCTGACCGTCTGCTGTCTCTGCAGGAACCTGAACACGCTGGCTGC  **>Me2G_2382**  ATGGGCTCTTTTGCACTGCCGGCGAACGCAAACACCCACGGTGGCTCTTTCCTGGCTGACCTGGAACCGAAAAGCTTCGCGGACGAATCTAAAGCAGTTATCGACTTCATTGCGGATTACTACAAGAACATCGAAAAATACCCGGTACAGTCTAAAGTAGAACCGGGCTACCTGTCTGCACAGCTGCCGGATACTGCTCCGCATAGCCCTGAATCCCTGGACGACATTCTGAAAGACGTGACCGACTCCATCATCCCAGGTCTGACCCACTGGCAGAGCCCGAATTTCTTCGGTTACTTCCAAGCGAACGCATCCACCGCGGGTTTCCTGGGCGAAATGCTGTGTAGCGGTTTCAACGTCGTCGGCTTTAACTGGCTGGCGTCTCCGGCAGCTACCGAACTGGAAAGCATTGTTATGGACTGGATGGGCAAAATGCTGAAACTGCCTTCTTCCTTTCTGTTCAGCGGTACTGGCGGTGGTGTCCTGCATGGCTCTACCTGCGAGTCCCTGGTATGTACCCTGGCCGCTGCGCGTGACGAGGCGCTGGAAAAACTGGGCGGCGGCTTCGATAACATCACTAAACTGGTCGTCTACGCGTCCGATCAGACCCACTTCGCTCTGCAGAAGTCTGCTAAACTGATCGGTATTCCACCAGCGAACTTCCGCCCGCTGTCTACCAGCTTCTCTACCGAATTTTCCCTGAGCCCGGATGCAGTGCGCGCGGCAATCGAAGATGATATCAAGAGCGGTTACGTTCCGCTGTACCTGTGCGCAACTGTGGGTACTACCGGCGCAGGTGCTGTGGACCCGATTGAGGAACTGGGTAAAATCGCCACCGAGTACAAACTGTGGCTGCACATCGACGCAGCTTATGCAGGTGGCGCCTGTATCTGCCCTGAGTACCGTCATTACCTGAATGGTGTTGAGCTGGCCGATTCCATCTCCCTGAACCCGCATAAATGGTTCCTGACCAACATGGATTGTGGCTGCCTGTGGGTTAAACACCCTTCCTTCCTGGTGGGTTCCCTGAGCACTAAATCTGACATCATGCGTAGCCGCTCTCCTGCATCCTCTACTAGCGCTAACGCGGCTCCGGTTATTGACTACAAAGACTGGCAAATCGCCCTGTCCCGTCGTTTTAAAGCCCTGAAGCTGTGGACTGTTATCCGTAAACACGGCTACTCTGGTCTGACGTACCATATCCGCTCCGACGTCAACATGGCCAAGCGTTTCGAAGCGATGGTGGCGAAAGATGAGCGTTTCGAAATTGCCGTCCCACGTAAATTCGCACTGGTTTGTTTCCGTCTGAAACCGAAACGTGAAAGCGAGGGCACTGAGCTGAACCGTAAGCTGCTGGATGCACTGAACGCTAGCGGTCGCGCGTTCCTGACTCAAGCGGTTCTGGGCGGTGTGTACGTGATTCGCTGTTCCATCGGTACCACCCTGACTCAAGATTCTCATGTGGATGACCTGTGGAAACTGGTGCAGGAGAAAGCGGATCGCCTGCTGTCTCTG  **>Me3G_0317**  ATGGGCTCCCTGACCTCTGATCTGGAACTGGAATACAATAGCAACAGCTCCATCAACCTGCTGGATTCTGAAGAATTTCGCCGCCAGGGTCACATGTTTGTTGACTTCATGGCAGAATACTATAAGACCATCGATAAATACCCGGTACGTTCTCAGGTTGAACCGGGCTACCTGAAAAAGCGCCTGCCGGAGTCTGCGCCGTACTCTCCGGAGTCTGTTGAAACCATCCTGGAAGACGTACAGGAGCACATTGTTCCAGGTATCACCCACTGGCAGAGCCCAAACTATTTCGCGTATTTCCCGGCTACTTCTTCTGTACCAGGTATTCTGGGTGAGATGCTGTCTTCCGGTTTCAACGTAGTGGGCATGGATTGGATCTCCTCTCCGGCAGCCGTAGAGCTGGAGAACATCGTTATGGACTGGCTGGGTCAGATGCTGAATCTGCCGAAATCTTTCCTGTTCTCTGGCAACGGCGGTGGTGTGATCCAAGGTACTACCTGTGAAGCACTGCTGTGTACTCTGACTGCGGCGCGTGACCGCGTACTGAAAAAGATCGGTCGTGAAAACATCAGCAAACTGGTTGTTTACTCTAGCGACCAAACGCACTGTGCGTTTCAGAAAGCAGCCCAGATCGTCGGTATCGATCGCAAAAACATCCGTGCTATCAAAACGACCAAGTCTTCTAGCTATGGTCTGAGCGCAGAATCCGTTCTGAGCCAGATCAACCTGGATGTGGAAGCTGGTCTGATCCCGCTGTTTCTGTGTGCCACCGTTGGCACGACCGCTATTACTGCTGTTGATCCTCTGCGTCCGCTGTGTGATGTGGCTAAACAATTCGGCATGTGGGTCCATGTTGATGCAGCATACGCTGGCTCCGCGTGTATCTGCCCGGAATTTCGCCACTTCATCGACGGCGTCGAGTGCGTGGATAGCTTCAGCTTCAACCCGCACAAATGGCTGTTCACCTCTCTGGACTGTTGCTGCCTGTGGGTGAAAGATCCATCCTGTCTGCTGTCTACTCTGTCCACCAACCCGCATTACCTGAAAAACGAGGCAACTGACCAGGAACCGAAGGGTCAGGAAGTAGGCTACAAAGATTGGCAGATCACGCTGTGCCGTCGTTTCCGTTCCCTGAAACTGTGGCTGGTCATCCGCAACTTCGGCGTGGCAAACCTGCGTAATTTCATCCGTAGCCACGTAGGCATGGCGAAGCTGTTCGAACGTCTGGTGCACAGCGATAAACGTTTTGAAGTCGCCTTCCCGCGTAACTTTTCTCTGGTCTGTTTCCGTGTCACTCCGTCTGCGGTTATGGATAAACTGCGTACGAAGTATAAAAACGGTACTCACGATCAATACCGTCAGCTGTCCGAGGAGGAGCGCACCAACGAATTCAATCGCGAACTGCTGGAGTCCATTAACGTGAGCGGTAAAGTGTTCATGACCCATGCGATTGTTGGTGGCATTTATGTTCTGCGTTTCGCTGCTGGCAACAGCATGACCATGGAAAGCCATGTACGTGAAGCCTGGACTGTGGTTCAGGAGCACCTGGAAGCGTTT  **>Me3G_1222**  ATGGGCTCCCTGGACTCTAACATGGCAGAAAGCGTACAGTTCAAACCGCTGGACGCGCAGGAATTCCGCAAACGTGCACACCAGATGGTTGACTTCATCGCGGACTACTATCAGAACATTGAGAGCTACCCTGTACTGACTCAAGTAGAGCCTGGCTTTCTGCGTAGCACGCTGCCAGACTCCGCCCCGCACCTGCCGGAGTCTTTCGAAACCATCCTGAAGGACGTTCAGAAAAAAATCATTCCGGGCATGACTCACTGGCTGTCCCCAAACTTCTTCGCGTTCTTCCCGGCAACTGTTTCTACCGCGGCCTTCCTGGGCGAGATGCTGTGCACTTGCTTCAACAGCGTCGGCTTCAATTGGCTGGCGTCTCCGGCATCCACGGAACTGGAGATTGTCGTGATGGACTGGCTGGCTAACATGCTGAAACTGCCGAAAACGTTTATGTTCTCTGGTACCGGTGGTGGTGTTATCCAGAACACCACGTCTGATTCTATCCTGGTGACCCTGATTGCCGCCCGTGACCGTGCGCTGCATGCTGTAGGCGCTCAAAACATGCACAAACTGGTTGTGTACGGTAGCGACCAAACCCATTCCACCTTTGCAAAAGCGTGCAAACTGGCGGGTATTTCTCCGGCGAATATCCGTGCGCTGCCTACCGGTATTGACGCCAACTTCTCTCTGTCTCCGCTGCTGCTGCGTCGCGCTGTTGAAGTAGATGTCGAGGCGGGTCTGGTTCCGCTGTTCCTGTGCGCAACCGTTGGTACTACCTCCACGACCGCAGTAGACCACATTGAACCGCTGGCCGAAGTGGCGAACGAGTATGGCATCTGGGTTCATGTAGATGCCGCCTACGCGGGCAGCGCCTGTATCTGTCCGGAATTTCGCCACTACCTGAACGGTATTGAACGCGTCGATTCCTTCTCCTTCTCCCCGCATAAATGGCTGCTGAGCTATCTGGACTGCTGTTGCCTGTGGGTGAAACAGCCGGGCCTGCTGGTTAAAGCGCTGTCTACTGACCCAGAATACCTGAAAAACAAGCCGTCTGAGAGCAACAGCGTAGTTGACTTCAAAGACTGGCAGGTGGGTACTGGTCGTCGTTTCAAGTCCCTGCGTCTGTGGATGGTGCTGCGTTCTTACGGTGTCGTAAACCTGCAGAACCACATCCGTTCCGATATCCGTCTGGCGAAAATGTTCGAAGGCTTCGTTAACTCTGACCCGCGCTTTGAAATTGTGGTACCGCGCCATTTCGCTCTGGTATGCTTTCGTCTGAACCCGTATCCGCAATGCTCCAGCGCGGATAACGAAATGCTGAACCGCACTCTGCTGGATTGGGTTAACTCTACGGGTCGCGTGTACATTACCCATACCATCGTGGGCGGTGTTTACATGCTGCGCTTCGCTGTTGGCGCAAGCCTGACCGATGAGCGTCACGTTGTTGCGGCATGGGAACTGATCGCTGAAGGTGCAGACCGCCTGCTGAAAGGCAGCCACTTC  **>Me3G_2284**  ATGGGCTCTCTGAACTCCGATCACGAACTGGAAACCTCTTCTGCAAGCTTCAATAACCCGATGGATAGCGAAGAATTCCGTCGTCAGGGTCATATGGTAATCGATTTCATTGCGGACTACTACCGTGATGTTGAAAAGTATCCGGTGCTGAGCCAAGTGGAGCCGGGTTATCTGCGCAAACGTCTGCCGGAGAGCGCTCCGTACAACCCTGAACCGATCGAAACCATCCTGCAGGATGTGCAGCAGCACATTGTGCCGGGTATCACTCACTGGCAGAGCCCATATTACTTTGCGTATTTTCCGTCCAGCGGTTCCATCGCCGGTTTTCTGGGCGAGATGCTGAGCTCCGGTTTTAACGTCGTGGGTTTCAACTGGATGTCCAGCCCGGCAGCCACTGAGCTGGAAAATATCGTCATGGACTGGCTGGGTGAAATGCTGAAACTGCCGAAATCCTTCCTGTTCAGCGGTACCGGTGGCGGTCTGATTCAGGGTACTACCTGCGAGGCGATCCTGTGTACGCTGGCGGCGGCTCGTGATCAGATTCTGAACGAAATCGGCCGCGAAAACATCTCCAAGCTGGTAGTGTACGGCAGCGATCAGACTCATTCTGCGCTGCAGAAGGGTGCACAGATTGCAGGTATTGACCCGAAAAACTTCCGTGCCATTAAGACTACGAAAAGCTCTAGCTTCGCTCTGACTCCGGAATCTCTGCAGGCTGCGATTGACCTGGACATCCAGGCTGGCCTGATTCCTCTGTTTCTGTGTGCGACCGTTGGCACCACTGCGATTACCACGGTAGACCCGCTGGGCCCTCTGTGCGAGCTGGCAAAACGTTACTCTATCTGGGTACACGTTGATGCAGCGTACGCGGGCAGCGCATGTATTTGCCCGGAATTCCGTCACTTTATCGACGGCATCGAAGGTGCTGACAGCTTCAGCCTGAACGCACACAAATGGTTCTTTACCACCCTGGACTGTTGCTGTCTGTGGGTCAAGAACCCGAACGCTCTGATCAAAGCCCTGTCCACTAACCCGGAGTTCCTGCGCAACAAAGCGAGCGATTCCAAACAGGTCGTTGATTACAAGGACTGGCAGATCACCCTGTCCCGTCGCTTTCGTGCTCTGAAGCTGTGGCTGGTACTGCGTAACTTTGGCGTGGCCAACCTGCGCAACTTCCTGCGCTCTCACGTAGGTATGGCACAACTGTTCCAGGAGCTGGTGGGCGGTGACAACCGCTTTGAGATCGTTGCGCCTCGTAACTTCGCAGTAGTTTGTTTCCGCGTTCTGCCAAGCGCTTCTGGCCTGGGTAACGGCAAAGCAAACGATGATGAACAGGAAGGCGCGAACGAACTGAATCGCAAGCTGCTGGTGTCTATTAACGCTAGCGGCCAGCTGTACGTTAGCCATGGCATGGCTACCGGTATGTACTTCATCCGTTTCGCAGTTGGCGCAACCCTGACTGAAGATCGCCACGTGATCGCAGCTTGGAAGGTAGTCCAGGAAAAGCTGGACGGTATTCTGACCACGTCC  **>Me4G_0535**  ATGGGTTCTCTGACTAGCGATCAACTGGACTGTAACTCTGGCCTGGTGGTAAACCCGCTGGACCCGGAAGAATTCCGTCGTCAGGCACACATGGTGATTGATTTCATCGCAGATTACTACAAAAACGTGGACAAGTATCCGGTTCGTTCCCAAGTTGAGCCGGGTTACCTGCGCAAATCTCTGCCGGAATGCGCTCCGTACAACCCGGAATCCATGGAAACTATCCTGCAGGACGTACAAGAGCACATCGTTCCGGGTATCACCCACTGGCAAAGCCCGAACTACTTCGCATATTTCCCGTCCAGCGGTTCTATCGCAGGCTTTCTGGGCGAAATGCTGTCTTCTGGTTTCAACGTAGTCGGCTTCAACTGGATTTCTTCTCCAGCGGCCACCGAACTGGAGAACGTTGTGATGGACTGGCTGGGTCAGATGCTGAAACTGCCAAAATCTTTCCTGTTTAGCGGCAACGGTGGTGGTGTAATTCAGGGTACTACCTGCGAAGCAATCCTGTGTACCCTGACTGCAGCTCGTGACCGTGTTCTGAACCGTATCGGCCGTGAAAACATCTCCAAGCTGGTCGTATACGGCTCTGATCAGACTCACTGCGCTCTGCAGAAAGCGGCGCAGATTGTGGGCATTGATCTGAAAAACTTTCGCGCAATTAAAACCACCAAATCCTCTTCTTACGGTCTGTCCCCAGACTCTCTGCTGACTCAGATTAACCTGGATGTTGAAGCCGGCCTGGTACCGCTGTTCCTGTGCGCAACGATCGGCACTACTGCGATCACTGCTGTAGACCCACTGAAACCGCTGTGTGACGTGGCAAAGCAGTTTGGTATCTGGGTACATGTTGACGCTGCCTATGCTGGTTCTGCATGTATCTGCCCGGAATTCCGCCACTTTATTGACGGTGTTGAAGGTGCGGATTCTTTCAGCCTGAACGCTCACAAGTGGTTCTTCACTACTCTGGACTGTTGTTGTCTGTGGGTTAAAGACCCGTCCGCCCTGGTAAGCTCTCTGAGCACTAACCCGGAATACCTGAAAAACAAAGCTACTGAGTCTAAACAGGTAGTGGACTACAAGGATTGGCAGATCACCCTGAGCCGCCGTTTTCGTTCCCTGAAACTGTGGCTGGTTATCCGCAATTATGGTGTGGCAAACCTGCGTCATTTCCTGCGTTCTCATGTGAACATGGCTAAACTGTTTGAACGCCTGGTAAGCTCCGACAAACGCTTTGAAATCGTGTTCCCGCGCCAGTTTTCCGTTGTCTGTTTCCGTGTATCCCCGTCCGCGGTCACGGACAAGCTGAAACCGAAAAACGGTAACTGCCATAGCCGTCAGCTGTCTGCAGAAGAAGAACCGATCAACGAATTCAACCGTGAACTGCTGGAATCTATCAACGCTTCTGGCAAAGCATACATGACCCACGCGGTCTTTGGCGGTATTTACGCCATCCGTTTCGCAGTTGGCGCAACTCTGACCGAAGAACGCCACACTATGGTTGCGTGGACGGCCGTGCAGGAACACCTGGAGGCGTTCCTGTCTACCAACACCAGCGTTAAC  **>Me5G_1302**  ATGGGCTCTCTGACTAGCGACCTGGAACTGAAATACAATAGCGACCTGCTGATCAATCCGCTGGACGCGGAAGAATATCGCCGCCAGGGTCACGTTATCATCGATTTTATCGCCGACTACTACAAAAACGTCGAGAAATATCCAGTTCGTTCCCAAGTTGAACCGGGCTACCTGCGTAAACGTCTGCCGGAGTCTGCACCGTACAATCCGGAGTCTATGGAAACCATTCTGCAAGACGTACAGGAGCACATTGTTCCGGGTATCACTCACTGGCAGAGCCCTAACTACTTCGCCTACTTCCCGTCTTCCGGTTCTATTGCAGGTTTCCTGGGTGAAATGCTGAGCTCTGGCTTCAATGTGGTTGGTTTCAACTGGATCTCCTCCCCGGCAGCTACTGAACTGGAAAATATCGTTATGGATTGGCTGGGTCAGATGCTGAAACTGCCGAAATCCTTCCTGTTCTCCGGTAACGGTGGTGGCGTTATCCAGGGCACTACTTGCGAAGCTATTCTGTGTACTCTGACCGCAGCTCGCGATCGTGTTCTGAACAAGATCGGTCGTGAGAACATCTCCCGTCTGGTTGTGTACGGTTCTGACCAGACTCACTGCGCGCTGCAGAAAGCCGCACAGATCGTAGGTATTGATGTGAAGAACTTCCGCGCGATTAAAACCACCAAATCCAGCTCCTACGGTCTGTCTCCGGACAGCCTGCTGGCACAGATCAACTCTGACGTTGAAGCTGGTCTGATTCCACTGTTCCTGTGCGCGACCGTTGGTACTACCGCGATCACTGCTGTTGACCCGCTGAAACCGCTGTGCGACGTTGCAAAACAGTTCGGCATCTGGGTTCATGTGGACGCTGCTTACGCGGGTAGCGCTTGCATTTGTCCGGAATTTCGTCATTTCATCGACGGCATTGAAGGTGCTGATTCCTTCTCCCTGAACGCGCATAAGTGGTTTTTCACCACCCTGGACTGTTGCTGTCTGTGGGTAAAAGACCCAAGCGCCCTGGTATCTAGCCTGAGCACCAACCCGGAATACCTGAAAAACAAAGCGACCGAATCCAAACAGGTTGTTGATTACAAAGACTGGCAGATCACCCTGAGCCGTCGCTTCCGCAGCCTGAAACTGTGGTTCGTGATCAAGAACTACGGTGTAGCAAATCTGCGCCACTTCCTGCGTAGCCACGTTAACATGGCAAAGCTGTTCGAGCGTCTGGTGGGTTCTGACAAACGCTTCGAGGTAGTCTTCCCGCGTCACTTCGCAGTTGCCTGCTTCCGTGTATCTCCGTCCGCGGTGACCGATAAACTGAAAACCAAGTACGAGAACTGCCTGCTGAGCGAGGAAGAGCAGATCAACGAGTTCAACCGCGAACTGCTGGAGAGCATCAACGCGAGCGGTAAAGCATACATGACCCACGCGGTAATGGGTGGTATCTACGCTATTCGTTTCGCTGCTGGCGCCACTCTGACCGAAGAACGTCACGTAATGGTTGCATGGACTGTTGTTCAGGAACACCTGGAGGCACTGCTGTCTGCTAGCGAACCG |
| --- |

**Table S11.** Summary of PAAS steady-state kinetic data with L-Phe as substrate.

| Enzyme | Km, mM | Kcat ×10-3, min^-1^ | Kcat/km ×10-3,  µM^-1^· min^-1^ | Kcat/Km of CK-PAAS, % |
| --- | --- | --- | --- | --- |
| CK | 1.13±0.31 | 0.96097±0.26 | 0.961 | 100 |
| Me1G_2960 | 1.7851±0.66 | 5.9878±2.32 | 3.3543 | 349.0426639 |
| Me2G_2379 | 3.7265±0.83 | 22.4±7.11 | 6.0129 | 625.6919875 |
| Me2G_2381 | 1.7943±0.60 | 6.7691±2.62 | 3.7725 | 392.5598335 |
| Me2G_2382 | 2.7762+0.51 | 7.8223±1.42 | 2.8177 | 293.2049948 |
| Me3G_0317 | 2.7821±0.73 | 7.5393±1.97 | 2.71 | 281.9979188 |
| Me3G_1222 | 9.9919±0.46 | 12.6±5.45 | 1.2614 | 131.2591051 |
| Me4G_0535 | 1.9977±0.54 | 5.087±1.31 | 2.5464 | 264.9739854 |
| Me5G_1302 | 9.8924±1.77 | 21.2±3.88 | 2.1442 | 223.1217482 |
| Me3G_2284 | 36.5853±4.35 | 34.6±12.63 | 0.94577 | 98.41519251 |

**Table S12.** The primers for pET28a vector construction.

| Gene name | Forward primer (5’-3’) | Reverse primer (5’-3’) |
| --- | --- | --- |
| Me1G_2960.1 | CGCGGATCCGAATTCATGGATGCCGAGCAATTGAGAG | TGCGGCCGCAAGCTTTTCTGTGGACAGCCTAGCTAAT |
| Me2G_2379.1 | CGCGGATCCGAATTCATGGGAAGTTTCGCTCTCCC | TGCGGCCGCAAGCTTGCATCTTGCATGCTCTGGCTC |
| Me2G_2381.1 | CGCGGATCCGAATTCATGGGAAGTTTCGCTCTCCCCG | TGCGGCCGCAAGCTTGCATCCTGCATGCTCTGGCTC |
| Me2G_2382.1 | CGCGGATCCGAATTCATGGGAAGTTTCGCTCTCCCCG | TGCGGCCGCAAGCTTCAATGACAACAGCCGATCAGC |
| Me3G_0317.1 | CGCGGATCCGAATTCATGGGGAGCCTCACATCCGATC | TGCGGCCGCAAGCTTAGAATGCTTCCAAGTGCTCTTG |
| Me3G_1222.1 | CGCGGATCCGAATTCATGGGTAGCCTTGATTCAAAC | TGCGGCCGCAAGCTTGAAATGGGAGCCTTTAAGCAAC |
| Me3G_2284.1 | CGCGGATCCGAATTCATGGGCAGCCTTAACTCCGATC | TGCGGCCGCAAGCTTAGATGTAGTTAGTATGCCATC |
| Me4G_0535.1 | CGCGGATCCGAATTCATGGGCAGCCTCACATCTGAC | TGCGGCCGCAAGCTTATTGACGGATGTATTTGTGG |
| Me5G_1302.1 | CGCGGATCCGAATTCATGGGGAGCCTCACATCCGATC | TGCGGCCGCAAGCTTCTAGGGTTCACTTGCACTTAG |
